# Small Angle X-Ray Scattering Studies of Mitochondrial Glutaminase C Reveal Extended Flexible Regions, and Link Oligomeric State with Enzyme Activity

**DOI:** 10.1371/journal.pone.0074783

**Published:** 2013-09-30

**Authors:** Magda Møller, Søren S. Nielsen, Sekar Ramachandran, Yuxing Li, Giancarlo Tria, Werner Streicher, Maxim V. Petoukhov, Richard A. Cerione, Richard E. Gillilan, Bente Vestergaard

**Affiliations:** 1 Department of Drug Design and Pharmacology, University of Copenhagen, Copenhagen, Denmark; 2 Department of Structural Biophysics, University of Copenhagen, Copenhagen, Denmark; 3 Cornell High Energy Synchrotron Source (CHESS) and Macromolecular Diffraction Facility at CHESS (MacCHESS), Cornell University, Ithaca, New York, United States of America; 4 Department of Chemistry and Chemical Biology, Cornell University, Ithaca, New York, United States of America; 5 European Molecular Biology Laboratory, Hamburg Outstation c/o DESY, Hamburg, Germany; 6 Center for Bioinformatics, University of Hamburg, Hamburg, Germany; 7 Protein Function and Interactions, Novo Nordisk Foundation Center for Protein Research, Copenhagen, Denmark; 8 Department of Molecular Medicine, Cornell University, Ithaca, New York, United States of America; University of Oulu, Finland

## Abstract

Glutaminase C is a key metabolic enzyme, which is unregulated in many cancer systems and believed to play a central role in the Warburg effect, whereby cancer cells undergo changes to an altered metabolic profile. A long-standing hypothesis links enzymatic activity to the protein oligomeric state, hence the study of the solution behavior in general and the oligomer state in particular of glutaminase C is important for the understanding of the mechanism of protein activation and inhibition. In this report, this is extensively investigated in correlation to enzyme concentration or phosphate level, using a high-throughput microfluidic-mixing chip for the SAXS data collection, and we confirm that the oligomeric state correlates with activity. The in-depth solution behavior analysis further reveals the structural behavior of flexible regions of the protein in the dimeric, tetrameric and octameric state and investigates the C-terminal influence on the enzyme solution behavior. Our data enable SAXS-based rigid body modeling of the full-length tetramer states, thereby presenting the first ever experimentally derived structural model of mitochondrial glutaminase C including the N- and C-termini of the enzyme.

## Introduction

Glutaminase C is known as an important protein in cancer related research [1]–[3]. Cancer cells have an altered glucose metabolism known as the Warburg effect. A critical feature of the changed metabolism is that pyruvate no longer enters the citric acid cycle, mandating a new source of metabolites to be formed [4], [5]. Glutaminolysis is a key hallmark of cancer cells where mitochondrial glutaminase (GA) catalyzes the conversion of glutamine to glutamate [6]. Important materials such as ATP and nucleotides are produced by further catabolism of glutamate in the Krebs cycle [5], [7], [8]. Glutaminase occurs naturally as two isoforms, namely a liver (LGA) and a kidney (KGA) form [9]–[11], as well as a shorter splice variant of KGA referred to as glutaminase C (GAC) [12]. KGA and GAC are over-expressed in many cancer cells but for breast, lung and prostate tumor cell lines only the GAC specie is found within the mitochondria [10]. The two kidney-type GAs are both phosphate activated enzymes but studies have shown that GAC has a much greater affinity than KGA towards glutamine at higher inorganic phosphate (Pi) concentrations [10], [12]. Because of GAC’s exclusive location and kinetic properties it has been suggested that this isoform is the key enzyme in mitochondrial metabolism in cancer cells, making it particularly interesting [10], [12]. Knowing the protein structure and its structural behavior in solution is an important step in understanding the mechanism of the GAC isoform and hence improve the understanding of cancer metabolism. It has long been thought that GAC forms a tetramer in order to exhibit activity but the activation mechanism *in vivo* is still not fully determined [13], [14]. Very little is known about the protein oligomerization states and structural changes in solution. However, recently published crystallographic X-ray structures, determined for a large fragment of the two kidney-type GAs, reveal the dimer- and tetramer-interfaces and most of the active site. Furthermore, it has earlier been shown that a change in the enzyme conformation occurs in the area of the tetramer-forming interface which keeps a proposed gating loop to the active site in an open conformation [15] and it has also been shown that Pi can bind in the active site [10]. The structure and mechanism of a large part of the N-terminal and the C-terminal remain unsolved. However, it has been suggested by several studies that significant functionalities reside at the termini [9], [10], [12], [15]. Here we elaborate on the understanding of the solution behavior of GAC by examining the oligomerization state of GAC in solution using small angle X-ray scattering (SAXS), analytical ultracentrifugation (AUC) and multi-angle light scattering (MALS) techniques to monitor the effect of Pi titration and increasing protein concentration. We show that the oligomeric state changes with concentration revealing equilibrium between a minimum of three species in solution. It was also shown that the formation of higher oligomers is more pronounced with addition of Pi. The study reveals great conformational freedom of the N- and C-termini of GAC and it was demonstrated that the C-terminal plays a role in the regulation and stabilization of the tetrameric state. We show a correlation between *in vitro* enzymatic activity of GAC and the oligomeric state. Furthermore, we present a SAXS-derived envelope of the full length GAC in the tetramer form, including the previously structurally unknown C- and N-termini. For the SAXS measurements a microfluidic setup for data collection was applied enabling screening of the solution behavior of GAC in response to different experimental conditions. Also, the use of the microfluidic setup enabled the study of a time-dependent oligomerization effect of the protein.

## Results

### Microfluidic Sample Environment

For the SAXS study, a microfluidic mixing setup was used. This provided an optimal experimental setup for screening the solution behavior of the two examined GAC constructs in response to relevant changes in the experimental conditions. The setup was originally developed within the BioXTAS project [16], [17] and was adapted for use on CHESS beamlines. In the CHESS design, the microfluidic chip was clamped with face-sealing o-rings to a water-cooled block containing a built-in boroscope for sample visualization [16], [17]. The new chip holders clamping technique made chip exchange and q-calibration much easier compared to earlier versions [16], [17] (Figure S1–S4 in File S1).

### Basic Observations from SAXS Data

Within the cell the first 72 residues of GAC are cleaved when GA has entered the mitochondria [9], hence we also analyzed GAC constructs starting from residue 73. SAXS solution measurements were thus performed from the following two constructs: GAC wildtype (residues 73–603; GACwt) and a GAC construct with a truncated C-terminal (residues 73–555; GACΔC). SAXS measurements at different concentration ranges with and without phosphate addition were carried out using the microfluidic mixing chip. Selected Pi titration SAXS measurements were performed with and without equilibration over time. Pi has been shown to activate the GAC enzymatic activity, and hence, change the oligomeric state. Measurements with and without equilibration time can potentially show the evolvement of the process. Based on the scattering data, it is possible to determine the average radius of gyration (R_g_) and average molecular weight (MW) of the species in solution. In monodisperse systems, it is also possible to derive the oligomeric state from this value, but for heterogeneous samples the R_g_ and MW values rather represent an average of all scattering particles present in solution. R_g_ and MW values are summarized in Table 1 together with the calculated theoretical values for each construct.

**Table 1:** Solution concentrations and basic biophysical parameters derived from the SAXS data.

			Estimated MW (kDa)			Estimated R_g_ (Å)		
		I(0)	Guinier App.	OLIGO	EOM	Guinier App.	OLIGO	EOM
**GACwt**	13.7	18.6±0.2	122	188	197	52±0.6	57	50
**conc (uM)**	22.2	44.9±0.2	181	205	204	54±0.3	61	52
	30.7	65.8±0.2	192	191	208	55±0.2	60	51
	39.3	88.6±0.2	202	210	245	56±0.2	62	58
	49.5	121.5±0.2	220	211	246	56±0.1	61	59
	58.1	143.8±0.3	222	215	230	56±0.1	62	57
	66.6	161.4±0.3	217	213	263	58±0.1	62	50
	73.4	175.8±0.3	214	212	262	58±0.1	62	59
**Phos.**	0	86.6±0.3	248	237		63±0.6	73	
**Conc.(mM)**	25	62.9±0.2	225	224		62±0.3	64	
	50	74.2±0.2	236	180		57±0.2	72	
	60	86.0±0.3	291	212		65±0.2	94	
	80	100.9±0.3	280	249		66±0.1	90	
	100	86.6±0.3	324	288		76±0.1	104	
**GACΔC**	24.5	42.0±0.2	169	210	204	54±0.3	65	49
**Conc(uM)**	33.9	63.1±0.2	184	212	176	53±0.2	66	48
	43.3	98.4±0.2	224	258	209	58±0.2	74	54
	54.6	138.3±0.2	250	286	208	59±0.2	78	55
	64.0	169.7±0.3	262	312	237	63±0.2	81	61
	73.4	199.1±0.3	268	329	255	63±0.1	81	62
	82.8	234.1±0.4	279	350	249	66±0.1	85	64
	97.9	269.2±0.4	271	365	256	69±0.1	87	66
**Phos.**	0	71.7±0.3	199	243		62±0.4	71	
**Conc.(mM)**	25	71.0±0.5	197	251		62±0.9	72	
	50	95.6±0.9	265	212		79±1.4	73	
	60	111.8±1.0	310	373		96±1.3	88	
	80	128.3±1.8	356	388		103±2.4	90	
	100	334.6±7.0	277	425		158±3.8	91	

Forward intensity scattering, the **I(0)** values, are estimated from data un-scaled for concentration. **Guinier Approximate MW (Guinier App** **MW)** and **Guinier App R_g_** are estimated from the forward scattering. **OLIGO MW** and **OLIGO R_g_** estimates are derived from OLIGOMER program analysis. **EOM MW** and **EOM R_g_** are estimates generated from EOM analysis. Theoretical MW for glutaminase C wild type construct is 234.20 kDa for tetramer and 117.10 kDa for dimer. Theoretical R_g_ is 57.50 Å for tetramer and 41.64 Å for dimer. The theoretical radius of Gyration for an octamer and a 16mer growing in an elongated direction is 94.7 Å and 186.5 Å respectively. Theoretical MW for glutaminase C construct with truncated C-terminal is 103.24 kDa for dimeric and 318.72 kDa for hexameric protein. R_g_ for GACwt and GACΔC are estimated to be very similar within the accuracy of SAXS data.

**Figure 1:**
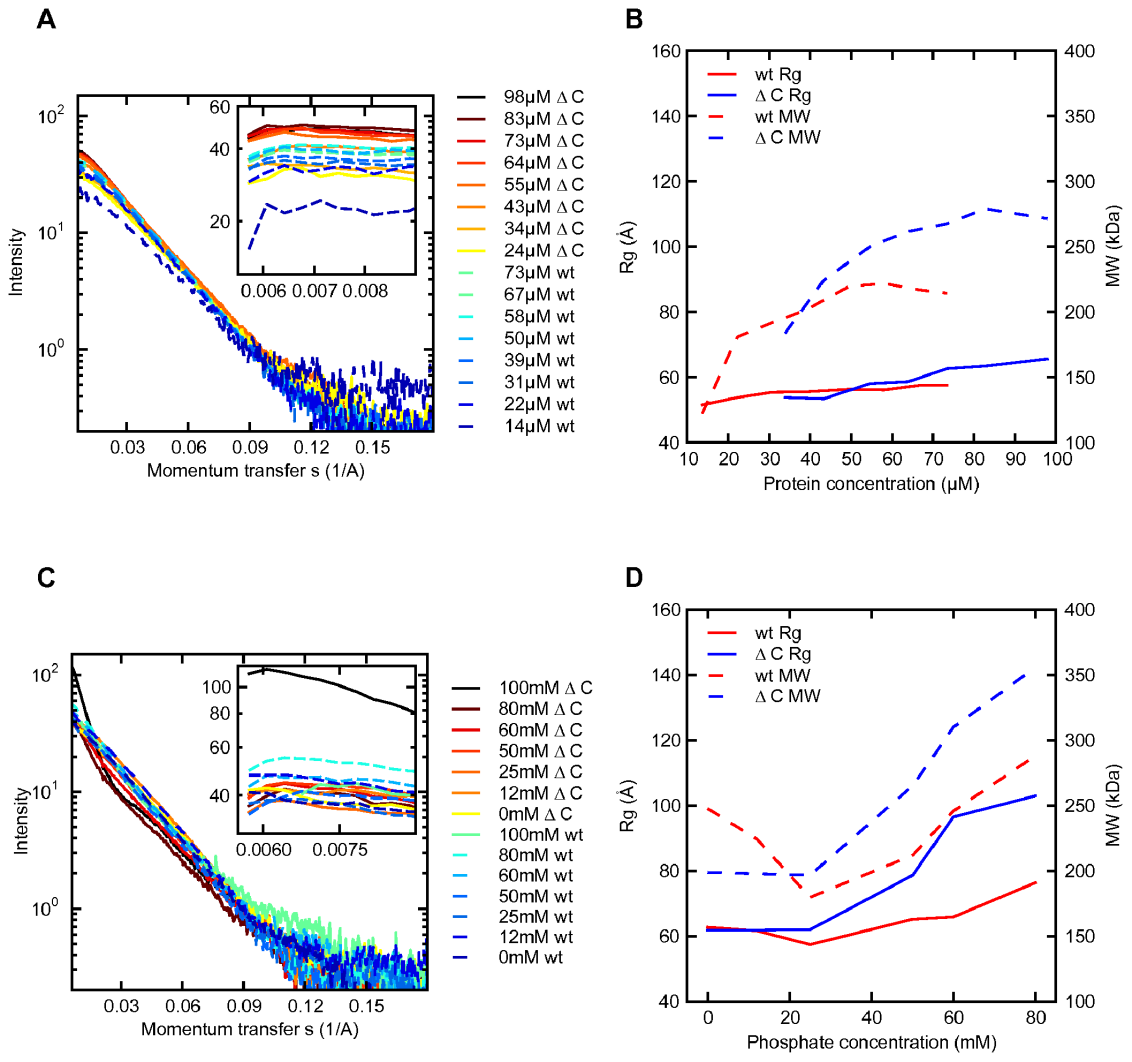
SAXS data and basic biophysical parameters. **a)** The microfluidic-mixing chip was applied to collect SAXS intensity curves for protein concentration dilution series of GACwt and GACΔC. The intensity curves plotted are background subtracted and normalized with concentration. The blue dashed lines show the data for the GACwt and the solid orange lines show the data for GACΔC. **b)** MW and R_g_ determinations from the SAXS data for the protein concentration screen data are plotted against protein concentrations. The solid lines show the R_g_values and the dashed lines show the MW values. **c)** The microfluidic-mixing chip was applied to collect SAXS intensity curves for phosphate titration series of GACwt and GACΔC. The protein concentration was kept constant at 30.7 µM for GACwt and the protein concentration was kept constant at 33.8 µM for GACΔC. The intensity curves plotted are background subtracted and normalized with concentration. The blue dashed lines show the data for GACwt and the solid orange lines show the data for GACΔC. **d)** MW and R_g_ determinations from the SAXS data for phosphate concentration screen are plotted against protein concentration.

When the protein concentration was increased for GACwt (GACwtCS) it is seen that the values vary from an average dimeric state, to beyond an average value of about 6 times the monomeric state (Table 1 and Figure 1a–b. See also Figure S5 in File S1 and see data from equilibrated samples in Figure S6 in File S1). The R_g_ and MW values are in continuous development with increasing protein concentration. It is hence concluded that the solution states investigated exist in a distribution between dimers and larger species, presumably tetramers and octamers.

It is also observed from the concentration screen of GACΔC (GACΔCCS) that the C-terminally truncated construct oligomerizes more extensively at higher protein concentrations than the wt protein (see Figure 1a–b, Table 1 and Figure S5 in File S1) suggesting that the C-terminal plays a role in the tetramer formation. This trend is even more evident in the results presented below.

When titrating Pi into the protein samples (GACwtPS and GACΔCPS), R_g_ and MW values evolve extensively, hence revealing further oligomerization (Figure 1c–d, Table 1 and Figure S5 in File S1). Again, the C-terminally truncated protein oligomerizes more extensively than the wt protein at otherwise comparable experimental conditions.

It is also seen that equilibration over time causes the oligomerization to proceed to even higher oligomeric states (Figure S6 in File S1). While the presence of even larger species in solution and hence an even more complex mixture of different species complicates detailed data analysis of these samples, the microfluidic setup has allowed detailed analysis of early equilibrium states.

### Rigid Body Modeling of the GAC Solution Structure

**Figure 2:**
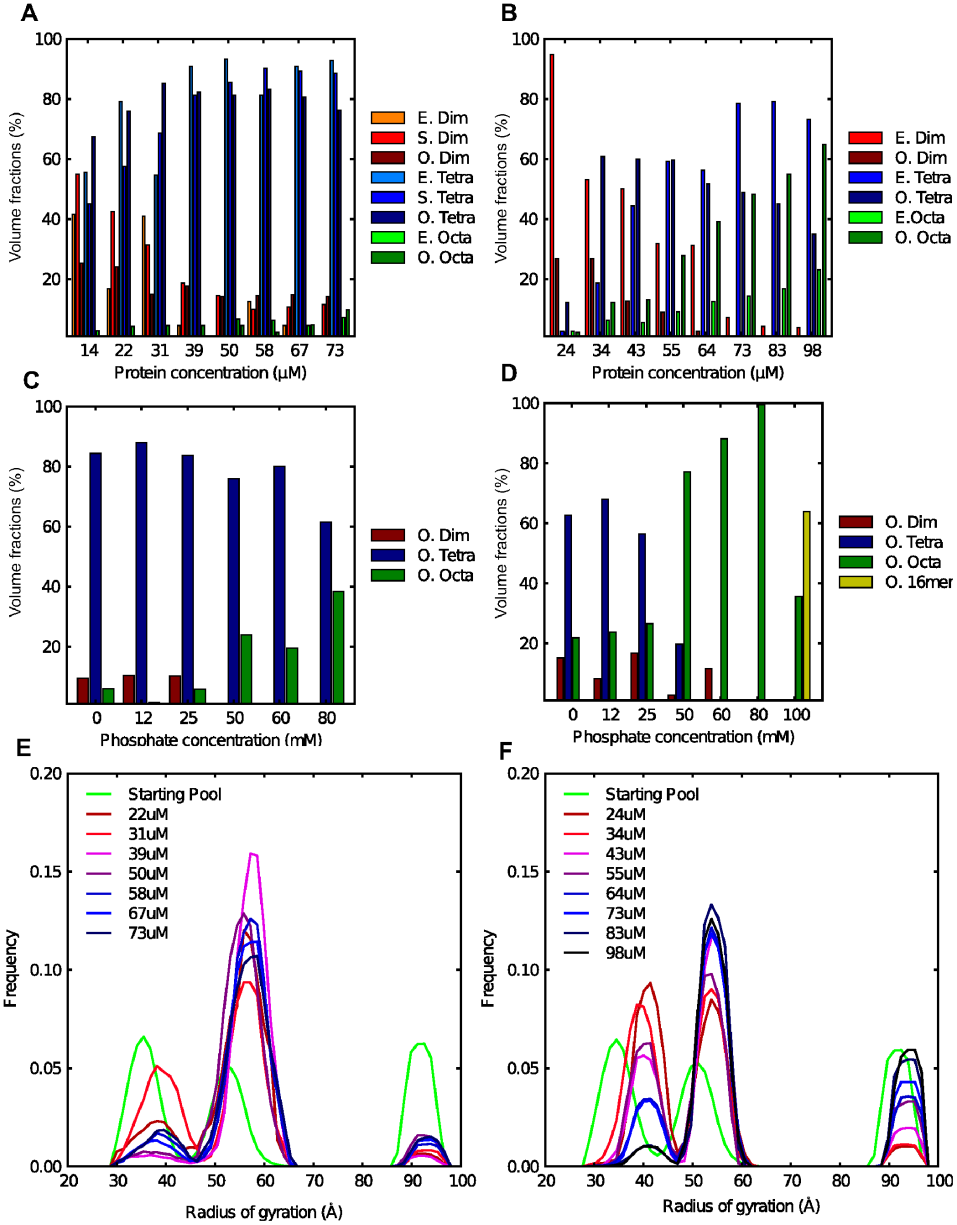
SAXS based analysis of solution systems flexibility and oligomeric states. a) Bar plot depicting the EOM estimated oligomer distribution (marked with E in legend), the SASRFMX distribution of dimers and tetramers (marked with S) and the OLIGOMER analysis estimated distribution (marked with O) of GACwt at the analyzed protein concentrations given on the x-axis in µM units. **b)** Bar plot depicting the EOM estimated oligomer distribution (marked with E) and the OLIGOMER analysis estimated distribution (marked with O) of GACΔC at the analyzed protein concentrations given on the x-axis in µM units. For a) and b) the EOM-derived distribution was estimated by taking the structures giving the best fit to the experimental curve. **c)** Bar plot showing the derived oligomer distribution given by OLIGOMER analysis as volume fractions for GACwt phosphate titration screen. **d)** Bar plot showing the OLIGOMER distribution as volume fractions for GACΔC phosphate titration screen. **e)** EOM analysis of concentrations screen of GACwt. R_g_ distribution of GACwt corresponding to the pool of structures (given pool of dimers, tetramers and octamers is shown in green). **f)** EOM analysis of concentrations screen of GACΔC. R_g_ distribution of GACΔC corresponding to the pool of structures (given pool of dimers, tetramers and octamers is shown in green).

**Figure 3:**
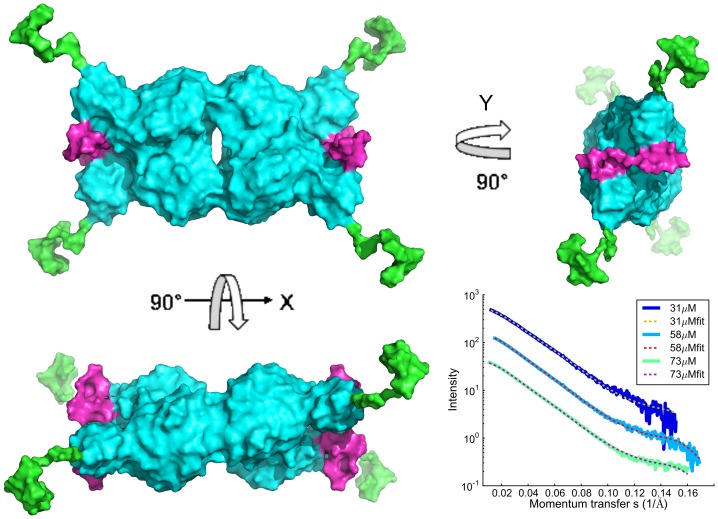
GlutaminaseC tetramer 3D low-resolution solution structure. Rigid body model of GACwt tetramer shown from three different orientations. The 3D structure was calculated with the SASREFMX program using a combination of the atomic resolution structure (pdb code 3ss3.pdb) and the GACwtCs data. The areas shown in pink and green are flexible regions and the rigid body model is therefore only an illustration of a structure that could typically be found in the solution. The plot in the right lower corner shows the SASREFMX fit to the experimental data for three of the in total eight scattering curves included in the calculation. The model can be compared to a model calculated while excluding the highest concentration data (Figure S7 in File S1).

The crystal structures of mouse GAC and human KGA (mGAC and hKGA) with and without substrate have recently been published [10], [15], [18]; however, it has never been possible to determine the structure of the N- and the C-termini. Here we have used SASREFMX [19] to identify if the known tetrameric structure is compatible with the solutions investigated in this study. In SASREFMX, polydisperse solutions containing partially dissociated assemblies, can be analyzed by simultaneous fitting of the scattering data from a concentration range of the protein species in equilibrium whereby the outcome is a rigid body model of the whole macromolecule and its volume fraction at each concentration [19]. From the R_g_ and MW values we estimated that the GACwtCS samples include mainly dimeric and tetrameric protein with the addition of higher oligomers in the most concentrated samples. Hence, the program was applied on this dataset both including and excluding the highest concentration measurements. The resulting tetrameric model including all data yielded chi values ranging from 1.1 to 2.9, whereas, by excluding the highest concentration, the highest chi value was reduced to 1.4. The typical models calculated in both scenarios were rather similar (see Figure S7 in File S1). The volume fractions of dimers and tetramers are shown in Figure 2a. The models calculated excluding the highest concentration data curves were preferred since the high concentration scattering curves could potentially include structural information that was not present in the lower concentration scattering curves. The low-resolution solution structure and fits to the experimental data for three of the seven included scattering curves are shown in Figure 3 (SASREFMX fits to all data curves included in the modeling are shown in Figure S13 in in File S1). Volume fractions and translations are listed in Table S1 in File S1 for both described models. See materials and methods for details.

### MALS and AUC Analysis of the Solution Oligomer Distribution

**Figure 4:**
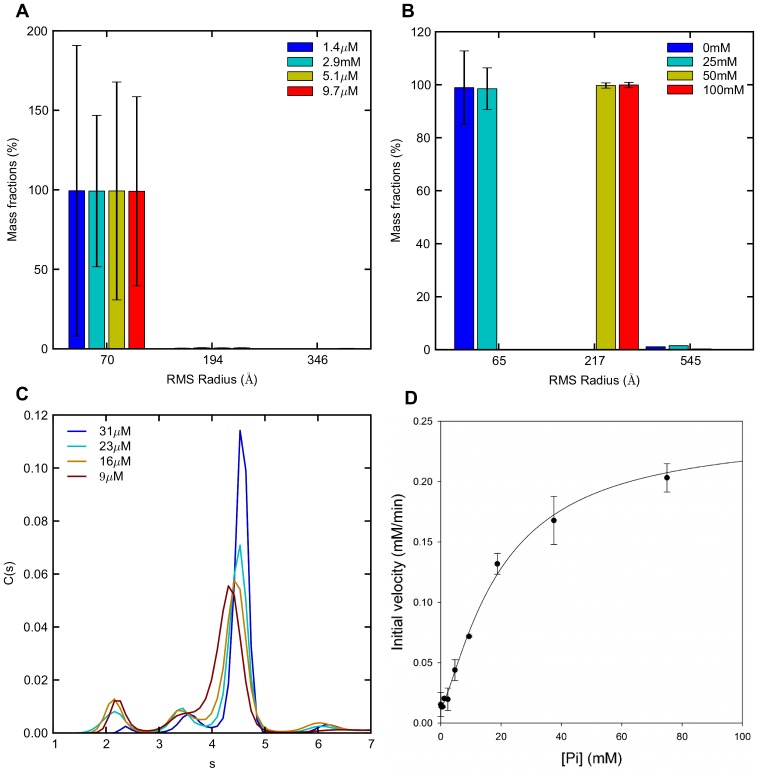
MALS and AUC based analysis of solution oligomeric state. **a)** MALS data for GACwt displaying mass fractions of oligomeric species in the protein solution in the protein concentration range 1.4 µM to 9.7 µM as detected on MALS with estimated R_z_ values (RMS radius) given on the x-axis. Elution concentration of the dominant peak is given in the legends. The error bars represent calculated fitting errors. **b)** MALS data for GACwt displaying phosphate dependent changes in protein oligomeric state. Mass fractions of species as detected on MALS with estimated R_z_ values given on the x-axis. Protein concentration was kept constant at 31 µM for all samples. The error bars represent calculated fitting errors. **c)** AUC sedimentation velocity data for GACwt construct showing a protein concentration dependent distribution of oligomeric states. The obtained continuous size distributions are plotted against the S values, corrected for buffer density and viscosity at 20°C (S_w20_), for the different protein concentrations as shown in the figure. **d)** Inorganic phosphate dependence of the activity of GACwt. The data is shown as mean +/− SD from three independent experiments. The line through the data points is drawn by inspection.

To verify the presence of different oligomeric species in equilibrium, as suggested by the basic SAXS analysis, we next subjected our proteins to MALS and AUC studies. Indeed the presences of several species were verified by both methods. Due to differences in experimental conditions when applying the different methods, results are not exactly quantitatively comparable. Within the concentration range, measurable by MALS (significantly lower than for the SAXS analysis for this protein system), three different species could be detected, but no significant changes in the distribution of oligomers were noticeable as a function of the protein concentration (see Figure 4a). Scattering corresponding to 99% of the volume fraction derived from a species with an RMS radius (R_z_) of approx. 70 Å, which could represent a tetramer. The theoretically calculated hydrodynamic radius (Rh) of a tetramer in solution is 77 Å; the data hence suggest that the protein largely exists in a tetrameric state in the investigated concentration range. The other oligomeric species present (from small volume fractions with estimated R_z_ values of 194 Å and 346 Å respectively) could indicate the presence of 16mers and higher oligomers (16mers theoretical R_g_ = 186.5 Å) forming by association in an elongated direction. The GACwt phosphate screen (GACwtPS); Pi concentrations from 0–100 mM, with an effective protein concentration constant at ∼5 µM, (see Materials and Methods for details) revealed a sudden increase in R_z_ after 25 mM Pi (see Figure 4b). Monomeric or dimeric protein are not detectable in any of the samples investigated, rather an intermediately sized species with R_z_ = 65 Å, i.e. in the vicinity of the theoretical Rh value for the tetrameric species (77 Å). Again a larger oligomer with R_z_ = 217 Å is detected, and a small amount of a multimer with R_z_ = 545 Å (MALS raw data are shown in Figure S8 in File S1).

Using AUC, a distribution between different oligomeric species is also observed, where the concentrations similar to those used for the SAXS experiments were used (See Figure 4c). The major peak observed at an S_w20_ (S value, corrected for buffer density and viscosity at 20°C) of approximately 4.5 S corresponds to a MW of a dimer. The smaller peaks observed at approximately 3.5 S and 6 S correspond to that of a monomer and tetramer, respectively. Expected S, R_g_ values and Rh values were estimated using the HYDROPRO program, confirming the presence of predominantly dimer species with smaller fractions of monomer and tetramer [20]. Furthermore, the predictions suggest that it is the more compact dimer (instead of a dimer forming in the elongated direction) that exists in equilibrium with tetramer and monomer in solution (See Figure S9 in File S1 for an illustration of a tetramer structure, the possible dimers and the estimated S, R_g_ values, and Rh values). As the protein concentration is increased the population of dimer increases significantly whereas only a small population of tetramer is observed also at higher protein concentrations (23 and 31 µM starting concentration). Hence, within the effective concentrations obtained during the sedimentation velocity experiments, mainly an monomer-dimer equilibrium is observed. Despite the fact that similar oligomeric species are observed when using AUC, the oligomeric distribution observed is different when compared to the other methods. A possible reason for this observation is that the time taken to perform the AUC experiments is much longer than for the other methods, allowing further aggregation of the tetramer into much larger oligomers. Alternatively, HYDROPRO fails to take into account the effect of the observed significant flexibility, yielding poor estimates of the theoretical S-values. Hence, the observed distribution of species in the AUC experiment may well be completely in accordance with the SAXS experiments. Changes were observed in the distribution of the three different species detected.

### SAXS Based Analysis of Dynamic Structural Ensembles and Oligomers in Solution

The SASREFMX rigid body modeling analysis confirmed that the previously known tetrameric structure with addition of the N- and the C-termini is supported by our SAXS data. Therefore, we proceeded to a more thorough approach for describing the flexible C- and N-termini using the program EOM [19], [21]. In accordance with experimental data, the program selects ensembles of theoretical scattering curves generated from very large pools of structures, where the assumed flexible parts of the protein are in random conformations. The selected pool of structures does not describe the actual combination of specific structures that is found in the solution, but rather a collection of structures representative of the solution state in size and conformation. As a complementary approach to the SASRFMX and EOM analysis we also employed the program OLIGOMER to evaluate the quaternary structure distribution [19], [22]. From a predefined set of structures of different size and shape, the program calculates a linear combination of the corresponding scattering curves, in accordance with the experimental data. This hence yields the volume fractions of each oligomeric species. It should be noted that, in contrast to the EOM and SASREFMX approaches, the OLIGOMER program only uses a few parameters for the best fit optimization to the SAXS data [19], [21] and suits here as a tool to cross-validate the results obtained with molecular modeling. The three different approaches to SAXS data analysis will in concert address the different degrees of freedom in the complex protein samples.

### Analysis of Ensembles and Oligomers in Solution for GACwt

According to the basic SAXS analysis, MALS and AUC analyses, a distribution of primarily smaller oligomers exists when screening the solution behavior of GACwt protein at different concentrations, however complemented by a small fraction of larger oligomers (Figure 2a, e, Table 1 and Figure S13 in File S1). Earlier reports based on TEM images [23] and our own TEM data (see Figure S10 in File S1) indicate that higher order oligomers form as elongated species. Hence, a tentative octamer and a hexadecamer model were constructed in accordance with what was learnt from the TEM measurements (see Figure S11 in File S1). Since the program EOM [19], [21] allows to model oligomers, we included dimers, tetramers and octamers in the starting pool. Good fits to the experimental data were obtained with chi values ranging from 1.2 to 1.7 (See Figure S12 and S13 in File S1). The selected ensembles containing dimers, tetramers and octamers suggest that compact conformations are never or rarely selected. It can hence be concluded that the samples contain significantly flexible and rather extended species of dimers, tetramers and octamers. Figure 2a depicts the relative distribution of dimers, tetramers and octamers as given by the EOM analysis (plots showing the distribution including standard deviation are shown in Figure S14 in File S1). Representative structures were carefully chosen (see Materials and Methods for details) among those most frequently selected by EOM and used as a starting pool for the complementary analysis with the program OLIGOMER for the GACwt data. The results obtained hence crosscheck the oligomer distribution given by EOM and SASREFMX. Within the given concentration range the analysis of GACwtCS data reveals a development in the distribution of oligomers where the level of tetramers increases with concentration up to 85% before the formation of octamers finally slightly depletes the tetramer pool. Accordingly, the presence of dimers decreases as the concentrations are increasing (Figure 2a and Table 1. See also Table S2 in File S1). Fits between the experimental data and selected EOM generated structures are shown in Figure S12 in File S1 and furthermore a plot is shown in Figure S13 in File S1 showing the fits obtained from EOM, OLIGOMER and SASREFMX analysis of the GACwtCs data. The chi values range from 1.3 to 2.3 (the highest chi values were found for the samples with the largest fraction of oligomers larger than tetramers). In the analysis no monomers are selected, consistent with the EOM result.

The overall parameters extrapolated using SAXS, MALS and AUC data from the GACwt phophate-screen revealed the presence of significant amounts of higher order oligomers and EOM analysis can therefore not be carried out. A manually built hexadecamer model was included in the OLIGOMER analysis. The analysis shows a shift in the solution distribution between 25 mM and 50 mM Pi, in accordance with the previous MALS analysis (Figure 2c and Table 1. See also Table S2 and Figure S12 in File S1). However, above 50 mM Pi increasing volume fractions of octamers are selected with a maximum of 38% at 80 mM Pi. The quality of the obtained fit to the experimental data decreases when octamer fractions are selected, indicating that other types of conformations could be present. The chi values range from 1.3 to 2.2.

### Analysis of Ensembles and Oligomers in Solution for GACΔC

The EOM analysis resulted in chi values ranging from 1.2 to 2.5 (Figure 2f, Table 1 and Figure S15 in File S1), with the discrepancies of the fits being most pronounced for scattering curves from high protein concentration samples (Figure S12 in File S1). Consequently a broad selection of R_g_ values is seen for dimers, tetramers and octamers, in the range of the larger R_g_ values within each random pool. Therefore, as it was observed for the wtCS data, it can be concluded that both dimer, tetramer and octamer conformations are significantly flexible and overall in an extended conformation. Also, the higher oligomer (octamer) conformation is only approximate (as seen by the increasing chi values at higher concentrations of Pi). The bar plot in Figure 2b depicts the percentage distribution of dimers, tetramers and octamers. A mix of dimers and tetramers is selected for the lower concentrations while above 64 uM mainly tetramers and octamers are selected. When compared to the results from GACwtCS analysis, GACΔC shows a significantly more rapid concentration dependent shift between the different oligomers. This is in line with the overall parameters extracted from the SAXS experimental curves.

The OLIGOMER analysis for GACΔC results in a continuous shift in the distribution of dimers, tetramers and octamers in the solution (See Figure 2b and Table 1. See also Table S2 in File S1). This confirms and elaborates on the result from the EOM analysis and from the basic SAXS analysis. The appearence of octamers starts at a lower concentration compared to what was seen for GACwtCS. Again, the chi values are high, and based on the MALS analysis, it is suggested that the discrepancies in the fits are due to the presence of even larger species in solution (see fits in Figure S12 in File S1).

During the phosphate screen the basic SAXS data analysis shows a rapid shift to very large oligomers. For the OLIGOMER analysis the earlier mentioned selected structures were included (the structures selected among the most frequent structures chosen by EOM in the GACΔCCS) (Figure 2d and Table 1. See also Table S3 in File S1). As for the GACwtPS a shift in the distribution is seen when increasing from 25 mM to 50 mM inorganic phosphate in the solution. Below 50 mM Pi primarily dimers and tetramers are found in the solution. Above that, octamers and 16-mers (and assumingly even higher oligomers) are present. This shift correlates very well with the shift seen in the parameters obtained from the basic SAXS analysis. When comparing the oligomer distribution derived from EOM analysis of the four datasets the results of the two pi screens seams more coherent than the results of the two concentration screens. As larger oligomers are induced by the presence of phosphate and hence larger changes in the oligomer distribution are seen over the analyzed Pi concentration ranges, we speculate that this enables the EOM program a more precise estimation of the oligomer content in the different the SAXS.

### Correlation of Protein Activity and Oligomeric State in Solution

The catalytic properties of GACwt and GACΔC were analyzed in correlation with the protein concentration. For most of the analyzed concentration range, the activity level of GACΔC was lower than that of GACwt. The glutamine hydrolytic activity of GACwt and GACΔC was measured as a function of increasing protein concentration. There was an increase in activity as the protein concentration was raised from ∼50 nM to 1 mM, and the activity of GACwt is consistently higher than that of the GACΔC construct. The trend seen for the GACwt construct is in agreement with the results obtained by Cassago *et al.* using a similar construct (aa 128–603) [10]. See Figure S17 in File S1.

The GACwt ability to catalyze L-glutamine conversion in correlation with the concentration of Pi was also studied. The data indicate that Pi induced oligomerization results in a tremendous increase in the activity of GAC, reaching a near saturation around 50 mM Pi. The activity of GACwt (50 nM) showed a substantial increase as the concentration of Pi was raised to 100 mM. The specific activity of the enzyme increased from 5 moles of glutamine hydrolyzed/sec/mole of enzyme in the absence of Pi to 70 moles of glutamine hydrolyzed/sec/mole of enzyme in the presence of 75 mM Pi. These findings are in good agreement with earlier published data by Cassago and co-workers for a similar construct used (aa 128–603), demonstrating that our construct (GACwt: aa 72–603) shows the same enzymatic behavior despite the elongated N-terminal [10], see Figure 4d.

## Discussion

By applying a microfluidic setup for the SAXS data collection, it was possible to screen the solution behavior of GAC in response to different experimental conditions. It is also possible to study solution behavior with SAXS using off-chip mixing and standard SAXS data collection, but here, in addition, we observe a time-dependent oligomerization effect. It is clear that the oligomeric state evolves over time, hence the effect of both protein concentration and Pi-concentration in particular are more easily analyzed from the microfluidic based data, compared to off-chip measurements. After equilibration over longer time-periods, the oligomeric state is so large that decomposition of the data into scattering data derived from smaller species is complicated or even impossible. This is evident also from the complementary data from AUC and MALS analyses.

MALS and AUC data were included in the study as a supplement to the SAXS data. Using these two techniques a distribution between a number of different oligomeric species were observed. The differences in the sizes of the oligomeric species present in solution, that are estimated by the different methods, can be understood when considering the experimental differences during the data collection. The MALS data confirmed the existence of a solution distribution between small and rather large oligomers. MALS is typically applied using protein concentrations that are significantly lower than those applied during SAXS, due to the dilution effect from the gel filtration integrated with the MALS technique. In the present study, the concentrations investigated were hence significantly lower during the MALS analysis than during the SAXS analysis. In either case MALS data reveal the presence of a relatively large fraction of a larger oligomeric species (assumingly tetramers in the concentration screen, and even larger species in the phosphate screen). Neither in the AUC nor the SAXS analysis, comparable fractions of very large species is detected. During the MALS analysis the protein experiences significant contact with very large surface areas on the column material. In some cases this is known to influence the oligomeric distribution, which hence may be the case here. In our SAXS analysis we clearly reveal that equilibration causes further oligomerization (Figure S6 in File S1), underlining the importance of applying a microfluidic setup for the SAXS data analysis. The shifts in oligomeric states in response to the experimental changes are very pronounced both in the SAXS and the AUC data. We hypothesize that the shift to lower oligomers is induced by the tetramer conformation being more prone to aggregation during the relatively lengthy AUC experiment, explaining the absence of significant amounts of tetramer in the AUC experiment. An alternative explanation would be that the calculated sedimentation coefficients only poorly reflect the experimental sedimentation of the highly flexible macromolecules. Hence, solution equilibria are potentially sensitive to the method specific experimental conditions during measurements, and also here the use of the microfluidic SAXS setup has played an important role. When applying SAXS analysis, the solution equilibria are not disturbed, since there is no physical separation of the individual oligomeric species (as is the case with both AUC and particularly MALS by centrifugation and chromatography respectively). Hence, SAXS enables analysis of the detailed differences between individual measurements in response to changes in the experimental conditions, which may be derived from the information rich data. In this study, the comparison of the solution state by applying three different methods, underscores the importance of using complementary methods for the analysis of complex solutes.

When comparing the actual estimates of R_g_ of individual oligomeric species from the different methods, there is a slight variation, but overall the obtained estimates agree surprisingly well. The clear observation from the SAXS data, revealing that the N- and C-termini of GAC are in random and extended conformations may explain why the different methods show slightly different estimates of the overall size of the molecules.

Importantly, however, in spite of differences in the quantitative estimate of species that exists as a distribution between different oligomeric species is confirmed by all three methods.

It can be questioned, whether the *in vitro* observations, revealing the existence of significant amounts of oligomers larger than tetramers (observed by TEM, SEC-MALS and SAXS) have direct biological significance. All three methods demonstrated pronounced oligomerization for both constructs, and relatively more so with addition of Pi. An earlier study used AUC to show that KGA forms tetramers and higher oligomers when Pi is added to the solution, in accordance with our results [24]. MALS and SAXS data analyses of the Pi titration are consistently showing a shift in oligomeric state at Pi concentrations above 25 mM, i.e. corresponding to high protein:Pi ratios. It has earlier been reported that enzymatic activity is sensitive to addition of Pi [10], [12], [25]. Our data elaborate on this observation, revealing a close connection between enzymatic activity, Pi concentration and the oligomeric state. The results evidently indicate a very low affinity to Pi. Exactly how Pi effects the oligomerization or the enzymatic activity and its biological relevance is not conclusive from this study. A recent study elaborating on the Pi effect on GAC structure has suggested that higher Pi concentrations could be expected in cancer cells and hence regulate GAC activity [10]. Also, it could be speculated that the proteins binding of Pi somehow changes the flexibility of the gating loop. Indeed, it has previously been shown that the enzyme changes conformation in the area of the tetramer-forming interface, keeping a proposed gating loop to the active site in an open conformation [15] and it has also been shown that Pi binds in the active site [10]. In addition, it has earlier been shown that KGA activity is regulated by phosphorylation [15]. A different study shows that GAC enzyme activity can be inhibited by binding of inhibitors to the dimer interface [25]. Likewise, as an alternative to the loop-regulation theory, it may be speculated, that the negatively charged inorganic phosphate occupies positive patches on the protein surface, thereby overall shielding for repulsions between positively charged amino acids which otherwise would diminish or prevent the dimerization and/or tetramerization. The most prominent site of tetramerization is the helix including amino acids 394–404 and indeed there are positively charged amino acids flanking both ends of this helix. Likewise, positively charged residues can be identified in the vicinity of the dimerization interface (e.g. Arg459 on one protomer, and Lys544 on the other). The suggestion that inorganic phosphate diminishes repulsive charge effects remains speculative, yet provides a plausible and simple explanation for the observations reported here.

As is observed from the SAXS data collection by applying the microfluidic setup, there is an observable time dependency on the oligomeric solution state. Hence, the ability to measure SAXS data at early time-points and thus prior to extensive oligomerization enables much more detailed analysis. This has made it possible to obtain a structural description of the different lower oligomeric states, together with a structural characterization of the N- and C-termini.

The three naturally occurring isoenzymes have a high sequence similarity, apart from differences in the distinct C-terminal sequences, suggesting significant functionality of the C-terminal [12], [14]. In this study our analysis of SAXS solution data revealed great conformational freedom of the N- and C-termini of GAC, hence also explaining why it has never been possible to obtain an atomic resolution structure including the termini [10], [15]. For reference, a secondary structure prediction of the N- and C-termini is shown in Figure S18 in File S1, demonstrating little prediction of secondary structural elements in accordance with our finding of structural flexibility in the termini. Importantly, we also showed that the C-terminal plays a role in the regulation and stabilization of the tetrameric state. This is evident, since the full-length construct showed a much stronger tetramerization tendency than the GACΔC construct. This makes it possible to also suggest which oligomeric state is the enzymatically active state. If the shift from lower to higher oligomers triggers enzymatic activity the GACΔC construct would show a higher catalytic rate than GACwt. However, our data showed that GACwt has an overall higher activity compared to the GACΔC construct. The tendency is most clear for the inorganic phosphate screen where GACΔC formed much higher oligomers compared to the wildtype construct. A construct similar to GACΔC (Δ539–603, GACΔC is Δ556–603) has been shown to have an increased Km compared to GACwt in the presence of inorganic phosphate [10]. Together, this indicates that the tetrameric state is more enzymatically active than the higher oligomers both in the presence and (to a lesser extent) in the absence of inorganic phosphate.

It has been suggested that the C-terminal of GAC plays a role in catalysis rate [10]. Likewise, GAC has a higher catalytic rate than KGA and the only difference between the two isoforms is the C-terminal. It has previously been speculated that the GAC N- and C-terminal may interact directly with the active site [25], [26]. Here, we show that the C-terminal is flexible *in vitro*, principally in accordance with this theory, since the flexibility may enable transient interaction with the site. It is also possible that transient interactions between two flexible C-termini provide an overall stabilization of the dimer, and it could even be speulated that the flexible tails partly prevent octamerization (based on the indication that octamers form in an elongating direction, see Figure S10 in File S1 for TEM images). These are, however, loose indications, and it remains elusive how the tetramer state would be stabilized by the C-terminal, which is placed far from the tetramerization interface. It has also been suggested that the N-terminal participates in transcription regulation and the terminal is shown to play an important role in protein structure and activity [9], [10], [15]. Again, the flexibility of the N-terminal that is demonstrated in this study is well in accordance with the existence of possible binding partners for regulatory purpose [27].

The N- and C-termini were shown to be highly flexible and rather extended. Hence, the presented rigid body model of the full length glutaminase C tetramer including the previously structurally undescribed C- and N-termini represents an average solution conformation, again emphasizing the flexible and extended nature of the N- and C-termini. For comparison, representative models generated by EOM both for GACwt and GACΔC are shown in Figure S16 in File S1. The selected models giving the best fit to the experimental data clearly reveal the high flexibility.

It should be questioned whether the *in vitro* observations of oligomers larger than tetramers (observed here by both TEM, SEC-MALS and SAXS) are directly relevant for *in vivo* conditions. In the living cell, the local concentration of GAC is expectedly lower than those applied in the *in vitro* measurements, while the overall concentration of macromolecules is significantly higher (the effect commonly referred to as macromolecular crowding). A range of specific protein partners may further influence the *in vivo* solution state. Hence, great care should be taken before directly *infering in vivo* relevance to the larger oligomeric species. However, it is very tempting to suggest that the qualitatively consistent observations from all applied methods (TEM, AUC, MALS and SAXS), clearly revealing that GAC oligomeric state is highly responsive to a number of experimental paramters (here: protein concentration, surface interactions, phosphate concentrations, presence/absence of C-terminal and equilibration time) has some functional relevance, also *in vivo*. It seems plausible that regulation of GAC activity involves changes in the oligomeric state, in response to *in vivo* conditions (such as local phosphate concentrations, presence of protein partners, phosphorylation and regulation of GAC expression level). We hence suggest that our observations of responsive changes in the oligomeric state reflects (one of) the *in vivo* regulation(s) of GAC enzymatic activity.

In conclusion, a structural solution description of the full-length tetrameric GAC enzyme, including the hitherto undescribed N- and C-termini are presented. This tetramer exists in equilibrium with both lower and higher oligomeric states, and the structural analysis has only been possible by applying a microfluidic solution SAXS setup, collecting SAXS data under numerous of experimental conditions. Our detailed analysis of the SAXS data are supplemented by both AUC and MALS data, hence providing a confirmation of the existence of different oligomeric states. From the SAXS data it is possible to link the observations between high concentrations of Pi and enzymatic activity, with the existence of tetramers in solution. Both protein concentration and the presence of inorganic phosphate directly influences the distribution of different oligomeric states in solution, and thus the enzymatic activity. We hence have provided extensive structural analysis of a highly complex enzymatic system, exhibiting a combination of both structural flexibility and oligomeric development.

## Materials and Methods

### Design of Microfluidic Mixing System

The measurements were carried out using a fully automated high-throughput microfluidic-mixing chip with a mixing design similar to the design described previously [16], [17]. The system facilitated automated exposures on samples whose buffer or concentration could be changed on the fly. A strong polystyrene (PS) to polymethylmethacrylate (PMMA) bonding procedure was developed to reduce the risk of leaks when utilizing the microfluidic chip. A novel holder was designed for the chip that not only ensured vacuum all the way to the exposure cell windows, but also incorporated a boroscope linked to a video monitor that made visual inspection of the sample possible during measurements. The sample temperature was regulated using a commercial chiller, which circulated water through the sample block. The microfluidic chips were sealed to vacuum using a special o-ring based clamping system. For detailed description of the mixing chip setup, the holder, performance and construction information, please refer to Figure S1–S4 in File S1. The microfluidic mixing setup, allowing rapid screens of a given protein in various buffer solutions, was used in order to perform screens of the GAC solution behavior.

### Glutaminase C Expression and Purification

A plasmid encoding mouse GAC (residues 73–603 for GACwt and residues 73–555 for GACΔC) were cloned into a pET28a vector and the proteins were expressed with an N-terminal histidine tag. The proteins were purified using a Ni-column, anion-exchange and gel-filtration chromatography. The tag was cleaved using thrombin after the Ni-column purification. Both constructs were kept in 20 mM Tris-HCl pH 8.5, 500 mM NaCl, 0.5 mM TCEP and 1 mM sodium azide for MALS, SAXS, and AUC measurements. For SAXS Pi titration experiments KH_2_PO_4_ (1 M KH2PO4, 20 mM Tris-HCl, 500 mM NaCl, 0.5 mM TCEP and 1 mM sodium azide; pH 8.4) was added to the buffer.

GACwt and GACΔC were both freshly purified prior to all experiments and the gel filtration elution buffer was used as blank. The solutions were kept cold at all times. For the SAXS measurements an initial concentration of 1.2 mg/mL and 1.4 mg/mL respectively were used.

### SAXS Data Collection

The microfluidic mixing setup was attached to the flight tubes on the beamlines providing vacuum all the way to the sample exposure windows. The system consumed roughly 30 µL sample volume per mixed sample. Series of samples at different concentrations with buffer measurements in-between were programmed in the control software for the microfluidic system and were performed automatically, including automated exposures. See File S1 for details.

The SAXS data collection for testing the performance of the microfluidic mixing chip and setup was carried out at F2 station of the Cornell High Energy Synchrotron Source (CHESS). F2 used an energy of 9.88 keV and provided a flux of 9×10^9^ photons/sec for a 250×250 µm beam. Samples examined at F2 were exposed for 40 sec with no signs of radiation damage. BSA (purchased from Sigma Aldric, USA) was dissolved in 50 mM HEPES pH 7.5 as done previously [28]. The protein solution was kept cold at all times and centrifuged at 14000 rev/min for 20 min prior to data collection.

A concentration screen of GACwt in the range 12 µM–73 µM and of GACΔC in the range 14 µM–98 µM was done. The Pi titration screens were carried out from 0–100 mM Pi for both constructs where GACwt protein concentration was kept constant at 30.7 µM and GACΔC protein concentration was kept constant at 33.8 µM. The SAXS measurements for GAC constructs were done at station G1 at CHESS. G1 used an energy of 9.86 keV and provided a flux of 3×10^∧11^ photons/sec for a 250×250 µm beam. Samples used at G1 were exposed for 20 sec. and showed no signs of radiation damage. Signs of aggregation were seen after 60 sec. Mixing times after addition of Pi or dilution of protein sample to start of measurement were 36 sec. for all measurements. Data reduction and background subtraction were done using the RAW data reduction software [29].

### SAXS Data Treatment

R_g_ and I(0) values were estimated using the program RAW [29] and the MWs were estimated using the forward scattering and BSA. When defining the Guinier range the s_max_*Rg values were always maintained in the range 1.28–1.31. All theoretical R_g_ values of 3D models used throughout the SAXS data evaluation for comparison with the experimental values were calculated using the CRYSOL program [30] from the ATSAS suite [19], [22], [31]. The theoretical R_g_ (57.5 Å) for the GACwt tetramer was calculated from the SASREFMX derived model. SAXS data were collected on a GAC construct mutated in the dimer-dimer interface, which traps the protein as a dimer (Y. Li, et al., in preparation) (a GACwt dimer). An R_g_ value of 41.64 Å was estimated from a scattering curve collected on this GACwt dimer construct. R_g_ values for the manually built octamer and 16mer are 94.7 Å and 186.5 Å respectively. R_g_ for GACwt and GACΔC octamers and 16mers are estimated to be very similar within the accuracy of SAXS data.

### Rigid Body Modelling

Rigid body modelling as performed using the recently developed program SASREFMX [19]. The method implemented in SASREFMX performs rigid body modeling of multisubunit complexes and oligomeric assemblies against the scattering data from polydisperse samples containing some amount of dissociation products. The optimized parameters in this case are the positions and orientations of the individual subunits (in terms of three Cartesian shifts and three rotation angles) as well as the volume fractions of the intact assembly and dissociation product(s). In the present study, the rigid portion of the GAC monomer was fixed in a position yielding the crystallographic tetramer upon application of the P222 symmetry. The arrangements of four sets of the N- and C-terminal portions in respect to the core were modeled in a symmetric way and the volume fractions of the tetramer and the dimer in solution were adjusted.

All eight scattering curves in the GACwtCS data set were applied. Furthermore, a scattering curve of the above mentioned GAC construct mutated in the dimer-dimer interface (the GACwt dimer) was used for the rigid body modelling and the tetrameric crystal structure of GACwt (3ss3.pdb) [10]. Default settings were used when running the program. During the analysis we calculated both models including all GACwtCS scattering curves, models excluding the GACwtCS scattering concentration curves at low concentrations and models excluding the high concentration GACwtCS scattering curves. Models were also calculated multiple times using different starting-points for the N-terminal and the C-terminal to reduce the effect of a particular random conformation. The models generated in individual runs demonstrated similar overall appearance.

### MALS Data Collection and Data Treatment

Purified protein was subjected to size exclusion chromatography (SEC) using a WTC-030S5 column (Wyatt Technology) equilibrated in 20 mM Tris-HCl pH 8.5, 500 mM NaCl (GF buffer) at a temperature of 23°C. For the concentration screen, K_2_HPO_4_ was absent, while protein concentration was varied within the range 1.4–9.7 µM. For the Pi screen, protein concentration was constant at 5 µM in the peak (injected concentration was 70 µM) while varying concentrations of K_2_HPO_4_ up to 100 mM. The size exclusion column was equilibrated in the GF buffers containing varying concentrations of K_2_HPO_4_ (0, 25, 50, and 100 mM). The SEC was coupled to a static 18-angle light scattering detector (DAWN HELEOS-II) and a refractive index detector (Optilab T-rEX, Wyatt Technology) was connected downstream of the SEC column. The SEC flow rate was 1 mL/min. Data was analyzed using the program ASTRA to obtain the RMS radius and mass distribution (polydispersity) of the samples. Monomeric BSA (Sigma) was used to normalize the light scattering signal and the refractive index values were used to obtain protein concentrations. RMS radius values plotted on the x-axis in Figure 4a–b were taken as an average of the detected RMS radius, with outliers excluded.

### AUC Data Collection and Data Treatment

Analytical ultracentrifugation experiments were performed on a Beckman XL-I analytical ultracentrifuge. Prior to loading of the protein samples, the centrifuge chamber, rotor and assembled cells were equilibrated at 4°C to minimize protein aggregation. All sedimentation experiments were performed at 4°C and the protein sedimentation was monitored at 280 nm at a rotor speed of 50000 rpm. The data was analyzed using a c(S) model implemented by SEDFIT [32]. The partial specific volume was calculated using Sednterp [33].

### Flexibility

Flexibility was assessed with Ensemble Optimization Method (EOM) [19], [21] which assumes coexistence of a range of conformations in solution for which an average scattering intensity fits the experimental SAXS data. A revised version of EOM [19], [21] was used to create a pool of 30,000 independent models for which the theoretical scattering curves are computed using CRYSOL [34] (exhibiting 10,000 dimeric, 10,000 tetrameric and 10,000 octameric conformations for the GACwtCS as well as for the GACΔCC). Afterwards, a genetic algorithm was used to select an ensemble of conformations with average theoretical profile fitting the experimental SAXS data. The genetic algorithm was repeated 100 times and the ensemble with the lowest discrepancy was reported as the best solution out of 100 final ensembles. In order to distinguish between EOM models that show dimeric, tetrameric and octameric oligomerization, an R_g_ histogram was calculated using all the models in the selected ensembles. Outcome R_g_ distributions for the selected ensembles were then compared to the R_g_ distribution of the pool in order to identity the oligomerization fraction.

EOM does not have any limitation of the linker length and thus the full flexible N- and C-termini were modeled. The 3ss3.pdb tetramer structure of GAC [10] was used as core. The 3ss3.pdb only has structural information for the amino acid sequence 145–549; therefore the rest of the structure was modeled by EOM as potentially flexible parts. The theoretical scattering curve was computed for each model in the pool by CRYSOL [34]. P222 symmetry was applied for the tetramer and the octamer was manually made considering a dimer and applying p222 symmetry. Afterwards, the genetic algorithm selected ensembles of a varying number of conformers (from 2 to 40) by calculating the average theoretical profile and fitting it to the experimental SAXS data. The genetic algorithm was repeated 100 times and the ensemble with the lowest discrepancy (chi) was reported as the best solution out of 100 final ensembles for each concentration in the series. EOM selected monomer, dimer and tetramer models are shown in Figure S16 in File S1.

### Oligomerization Analysis Performed Using the Program OLIGOMER

The scattering profile from an equilibrium mixture without inter-particle interactions is a linear combination of the scattering intensities of individual components, weighted by their volume fractions *ν_k_* [30]:
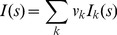
(1)


If the scattering patterns of the mixture components *I_k_* are available (or if their models are known), the values of *ν_k_* can be directly computed from the SAXS data by the program OLIGOMER [35], using a linear least squares fitting. In the present study OLIGOMER was applied to find the volume fractions of the dimer, tetramer and octamers (where applicable).

In all SAXS-based modeling approaches presented here an appropriate scaling of the predicted intensity curve is performed to yield the best agreement with the experimental data minimizing the discrepancy χ:
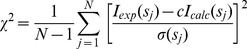
(2)where *c* is a scaling factor, *N* is the number of points and *σ* denotes the experimental errors. Optionally, a background constant may also be adjusted to provide better fit at higher angles. All four datasets presented in the paper were analyzed (GACwtCS, GACwtPS, GACΔCCS and GACΔCPS) and applied on the most frequent models selected by EOM. From each EOM run the 10 most often selected models were chosen, generating a pool of 80 models for GACΔC and 70 models for GACwt. Among these structures the most typical structures were choosen to respresent a broad range of R_g_ values (both extended and more compact structures). For GACΔC, the R_g_ values for dimers included in the analysis were 38.5 Å, 45.0 Å and 40.2 Å. The R_g_ values for tetramers included are 55.6 Å and 45.2 Å. For GACwt, the R_g_ values for dimers included in the analysis were 43.7 Å, 41.1 Å and 32.3 Å. The R_g_ values for tetramers included were 62.8 Å and 65.0 Å. Also, manually built octamer (R_g_ = 91.4 Å) and 16-mer structures (R_g_ = 157.1 Å) were included in the analysis. The octamer and 16-mer are shown in Figure S11 in File S1. OLIGOMER program was always run including the possibility for background constant adjustment. All constants derived upon analysis of the four datasets remained in the volume fractions range 0.000–0.008.

### GAC Activity Assay Protocol

The activity of GAC towards glutamine was measured by a two-step coupled assay. In the first step, GAC was added to a buffer (65 mM Tris-acetate (pH 8.6) and 0.2 mM EDTA) containing 20 mM glutamine and various concentrations of K_2_HPO_4_. This mixture was incubated at RT for 10 min. and the reaction was quenchd by addition of ice-cold hydrochloric acid (HCl) to a final concentration of 0.3 M. An aliquot of this was added to a buffer (160 mM Tris-HCl (pH 9.4)) containing 0.35 mM adenosine diphosphate, 1.7 mM nicotinamide adenine dinucleotide (NAD) and 6.3 U/mL glutamate dehydrogenase and incubated at RT for 50 min. Subsequently, the absorbance at 340 nm was measured and converted to glutamate concentrations using the extinction coefficient for the conversion of NAD to NADH of 6220 M^−1^cm^−1^.

## Supporting Information

File S1 Supporting information figures and tables.
(DOCX)
