# Correction: Small Angle X-Ray Scattering Studies of Mitochondrial Glutaminase C Reveal Extended Flexible Regions, and Link Oligomeric State with Enzyme Activity

**DOI:** 10.1371/annotation/80d64df5-065e-4be9-a4e8-31c4cba67114

**Published:** 2013-12-10

**Authors:** Magda Møller, Søren S. Nielsen, Sekar Ramachandran, Li Yunxing, Giancarlo Tria, Werner Streicher, Maxim V. Petoukhov, Richard A. Cerione, Richard E. Gillilan, Bente Vestergaard

The name of the fourth author was spelled incorrectly. The correct name is: Yunxing Li. 

