# Supplementary material for: Small Angle X-Ray Scattering Studies of Mitochondrial Glutaminase C Reveal Extended Flexible Regions, and Link Oligomeric State with Enzyme Activity
Source: PLoS One. 2013 Sep 30;8(9):e74783. doi: 10.1371/journal.pone.0074783 (PMC3787022; doi:10.1371/journal.pone.0074783)
Supplement: File S1 — Supporting information figures and tables. (DOCX) [file pone.0074783.s001.docx]

- **Supplementary Information:**

**
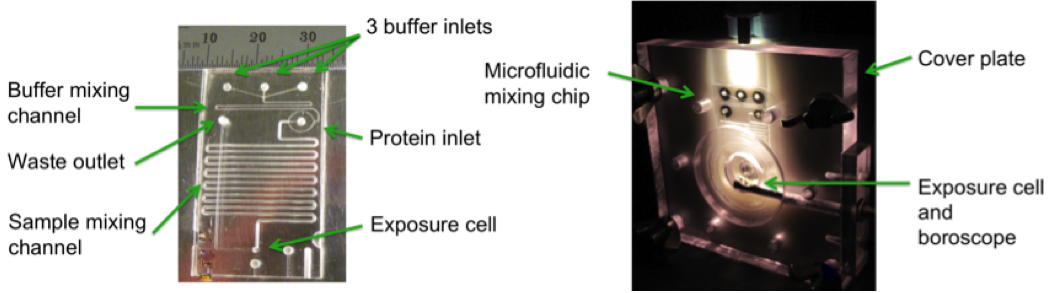
**

**Figure S1: Photo of the microfluidic chip.** Photo of the microfluidic mixing chip (left), chip holder, chip and boroscope (right).

**Microfluidic chip fabrication:**

A 1 mm thick, 4 cm x 2.5 cm wide piece of PMMA was used for the microfluidic chip. 125 µm thick Polystyrene (PS) was used as window. The chip channel design was identical to the one presented by Toft and co-workers in 2009.

The chip features three buffer inlets, a buffer mixing channel, a protein solution inlet and a sample mixing channel. The buffer mixing splits into two channels and merges with the protein inlet to laminate the protein sample on both sides. Three buffer components can thus be mixed with a protein solution and the sample transported to the sample exposure cell. The sample exposure cell is 1 mm deep and 1 mm wide corresponding to 0.79 μl of exposed sample volume. See figure S1.

The channels were machined using micromilling technique. The channel width for the buffer channels was 100 μm, the buffer mixing channel 200 μm, and the sample mixing channel 500 μm. All channels were 100 μm deep. The length of the mixing channels was based on a flow rate of 0.6 μL/min and a diffusion coefficient of buffer and protein of 10^-5^ cm^2^/s and 10^-7^ cm^2^/s respectively [[1](#_ENREF_1)].

A chip holder was designed to enable in vacuum chip mounting, cooling of the chip environment down to 4°C, and to enable a view of the sample chamber through a boroscope.
See figure S2. was used for the analysisant 1, 2 and 3), two tetramer conformations (variant 1 and 2) and one octamer conformat The holder, machined from polyethylene, has two halves. The half upstream of the x-ray path has a small indent for mounting the chip while the downstream half clamps the chip in place. The holder is fixed to an xz-stage to allow accurate positioning in the x-ray beam. Both parts are attached to the flight tube of the instrument by KF-40 vacuum bellows. See Figure S2 for an overview. The Inlets were attached to Hamilton glass syringes on syringe pumps (New Era Pump Systems, NE-500), which were remote controlled by custom made software similar to the automation software described earlier [[1](#_ENREF_1)].

The chip mount had 4 threaded holes drilled into it on top and channels leading down to the inlets of the microfluidic chip. Cooling was achieved by passing chilled antifreeze solution at 0°C through a 1.5 cm diameter channel parallel to the back of the chip.

**Figure
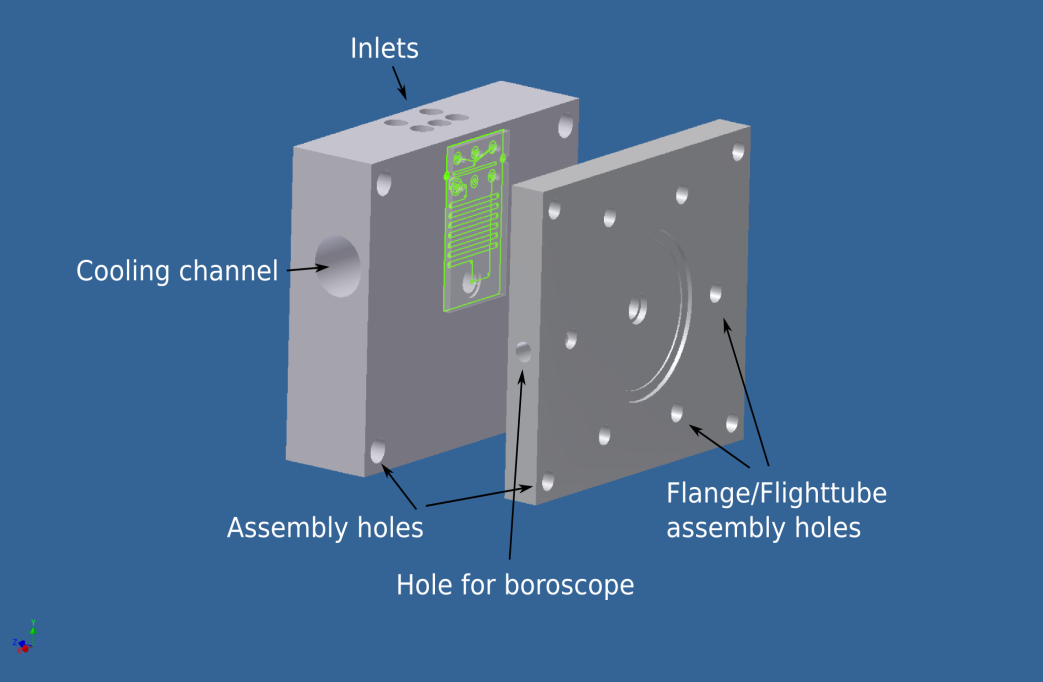
S2: Drawing of the chip holder assembly.**

**Bonding procedure:**

To obtain good bonding between the PS and the PMMA, the following procedure was used: to prepare the PS windows for bonding, each piece was cleaned first with water, then EtOH, dried with N_2_, plasma cleaned for 5 min. on both sides, cleaned with EtOH again and finally dried with N_2_. The PMMA was prepared by soaking for 10 min. in a 50 % isopropanol ultrasonic bath, drying with N_2_, baking for 10 min. at 90 °C, plasma cleaning for 20 sec. on both sides (Harrick Plasma Cleaner, 30W applied to the RF coil), cleaning with 50 % isopropanol and finally drying with N_2_.

The PMMA and PS windows were aligned and pressed together between two silicon wafers in a heat press. The temperature was set to 45 °C until the pressure had reached 400 lbs. The temperature was then increased to 95 °C and pressure was kept for 45min. The temperature was then set to 45 °C and the press was left to passively cool down to 45 °C which would usually take 3-4 hours after which the pressure would be released very gently.

-
- **a)**
-
- **b)**
- **Figure S3:** **Microfluidic setup reproducibility and accuracy of the mixing system.** BSA dilution series mixed either on the chip or on the lab bench, N=3. In both cases SAXS data were collected using the chip. a) SAXS intensity curves for six dilution series were recorded. b) The forward intensity scattering I(0) is proportional to the concentration over the range studied. Chipmixed: R^2^=0.97, 2-9 % reproducibility error at 95 % confidential level. Handmixed (mixed on lab bench): R^2^=0.99, 1-7 % reproducibility error at 95 % confidential level. A factor of 0.033 was found differences between expected and actual concentration when mixing on the chip.

**a)**

**b)**

**Figure S4: Chip sample consumption.** For changing from buffer to protein sample 30.25 μl is needed. For changing from protein to buffer 38.5 μl is needed. Difference curves are plotted in the figure in which a reference SAXS intensity curve is subtracted from the SAXS intensity curves obtained during sample injection (N=2). **a)** Starting with a buffer solution filled sample chamber, BSA solution was continuously injected in the microfluidic cell at a 5 μl/min flow rate for 44 μl. The 44 μl intensity curve was used as reference. **b)** Starting with a BSA solution-filled sample chamber, buffer solution was continuously injected in the microfluidic cell at a 5 μl/min flow rate for 55 μl. The 55 μl intensity curve was used as reference.

**Figure S5: SAXS data plotted with error bars. a)** The intensity profiles of a concentration screen of GACwt are background subtracted and normalized for concentration. **b)** The intensity curves plotted of a concentration screen of GACΔC are background subtracted and normalized with concentration. **c)** The intensity curves plotted of a phosphate screen of GACwt are background subtracted and normalized with concentration. **d)** The intensity profiles of a concentration screen of GACΔC are shown, background subtracted and normalized for concentration. The BSA SAXS curve used to estimate the MW of the GAC SAXS data are in addition plotted as normalized with concentration for each of the four screens (shown in purple).

**a)**

 **b)**

- **Figure S6: Scattering curves for inorganic phosphate titration with equilibration time.** The microfluidic-mixing chip was used to collect SAXS intensity curves for inorganic phosphate titration series of GACwt and GACΔC. The intensity curves plotted are background subtracted and normalized with concentration. The dashed lines show data that were given 30 min. equilibration time. The solid lines show data that were given less than 1 min. equilibration time. **A)** Blue lines show the data for GACwt construct. The red and orange dashed lines show the data for equilibrated samples. **b)** Orange lines show the data for GACΔC. The data of the equilibrated samples are shown in black and orange (dashed lines).


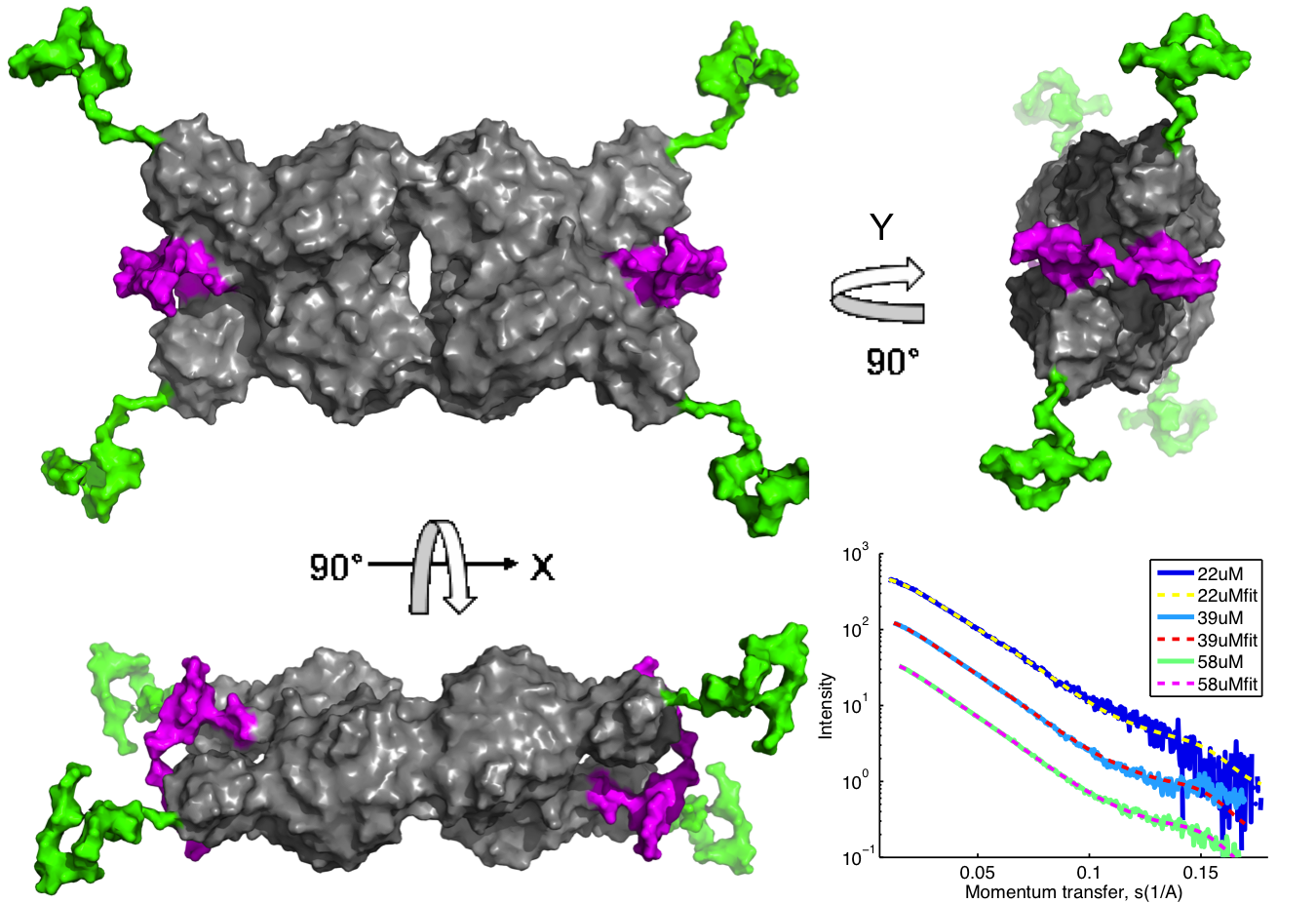


- **Figure S7: Glutaminase C tetramer 3D low-resolution structure.** Rigid body model of GACwt tetramer shown in three different orientations. The 3D structure was calculated using the program SASREFMX using a combination of the atomic resolution structure (pdb accession code 3ss3.pdb) and the GACwt concentration series data. Only six of the total eight scattering curves in the GACwt concentration series were used. This structure can hence be compared to the model obtained when including all data from the concentration scan (see main manuscript, Figure 3). The areas shown in pink and green are flexible regions, and it is therefore only an illustration of a structure that could typically be found in the solution. The plot in the right lower corner shows the SASREFMX fit to the experimental data for three of the eight scattering curves included in the calculations.

| Model | Protein concentration of scattering curves | | | | | | | |
| --- | --- | --- | --- | --- | --- | --- | --- | --- |
| Full dataset | 14 uM | 22 uM | 31 uM | 39 uM | 50 uM | 58 uM | 67 uM | 73 uM |
| Tetramer Vol. fraction in % | 48.5±5.6 | 61.0±5.5 | 72.5±5.7 | 85.3±6.1 | 88.4±4.5 | 94.3±9.2 | 89.3±5.7 | 88.5±5.8 |
| Dimer Vol. fraction in % | 51.5±5.6 | 39.0±5.5 | 27.5±5.7 | 14.7±6.1 | 11.6±4.5 | 5.6±9.2 | 10.7±5.6 | 11.5±5.8 |
| Reduced dataset | 14 uM | 22 uM | 31 uM | 39 uM | 50 uM | 58 uM |  |  |
| Tetramer Vol. fraction in % | 46.3±3.7 | 59.2±4.0 | 70.4±4.2 | 83.6±4.5 | 87.0±3.3 | 92.7±4.7 |  |  |
| Dimer Vol. fraction in % | 53.7±3.7 | 40.8±4.0 | 29.6±4.2 | 16.4±4.5 | 12.9±3.3 | 7.3±4.8 |  |  |

**Table S1.** Volume fractions with error bars of tetramer and dimer obtained from rigid body modeling using SASREFMX. For the model including all eight GACwtCS data curves (full dataset) the obtained translations are x: -26.8 ± 18.8  y: -49.0 ± 13.8   z: 90.6 ± 2.3 (N=4). For the model including six GACwtCS data curves (reduced dataset) the obtained translations are x: -19.8 ± 6.1 y: -53.6 ± 4.8  z: 90.0 ± 2.8 (N=8).

**A**


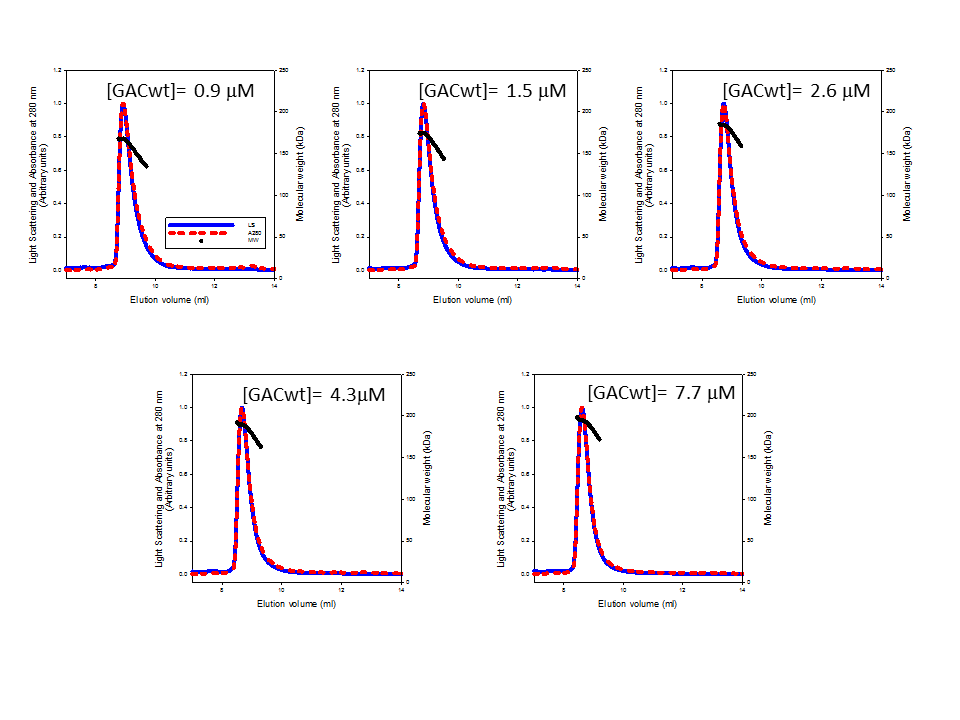
**B**


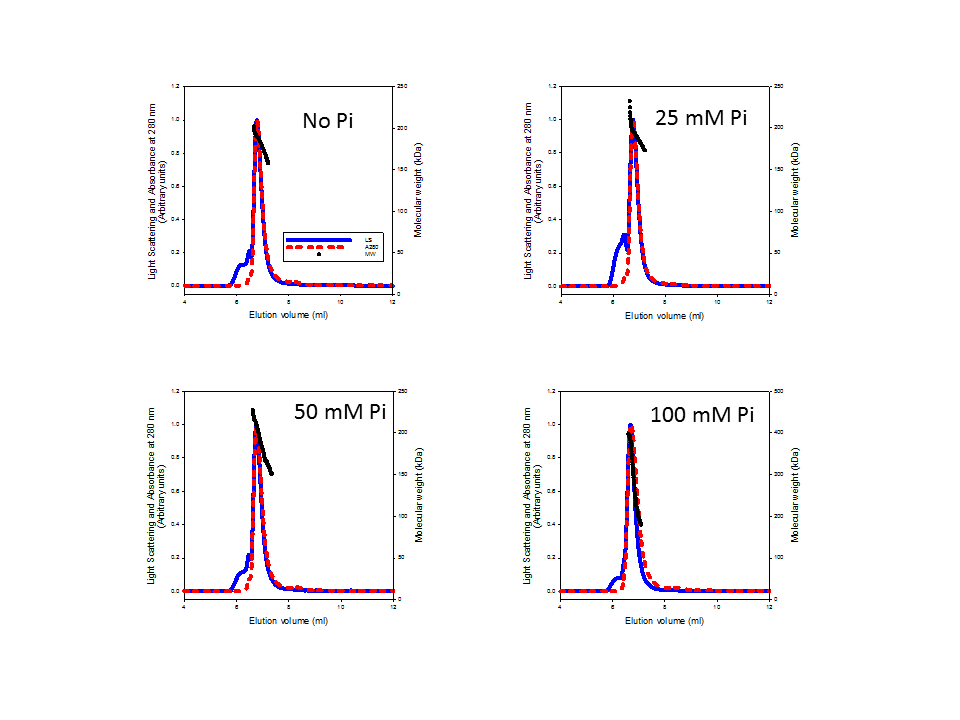
 **Figure S8**: **a)** MALS profile for GACwt at varying concentrations of protein. The plots show Rayleigh light scattering detected at a 90 ^o^ detector (continuous blue line), absorbance at 280 nm (dashed red line) and the MW for individual slices across the peak (black filled circles). **b)** MALS profile for GACwt in the presence of varying concentrations of Pi. The plots show Rayleigh light scattering detected at the 90 ^o^ detector (continuous blue line), absorbance at 280 nm (dashed red line) and the MW for individual slices across the peak (black filled circles).

| Oligomer | S | R_g_ (Å) | Rh (Å) |
| --- | --- | --- | --- |
| Monomer | 3.72±0.27 | 33.5±0.02 | 39.5±0.01 |
| dimers |  |  |  |
| red-green (compact) | 5.25 | 41.9 | 54.2 |
| black-cyan (compact) | 5.76 | 41.3 | 54.2 |
| black-green (elongated) | 4.93 | 53.8 | 57.2 |
| red-cyan (elongated) | 5.46 | 51.6 | 56.9 |
| tetramer | 7.90 | 57.0 | 77.2 |


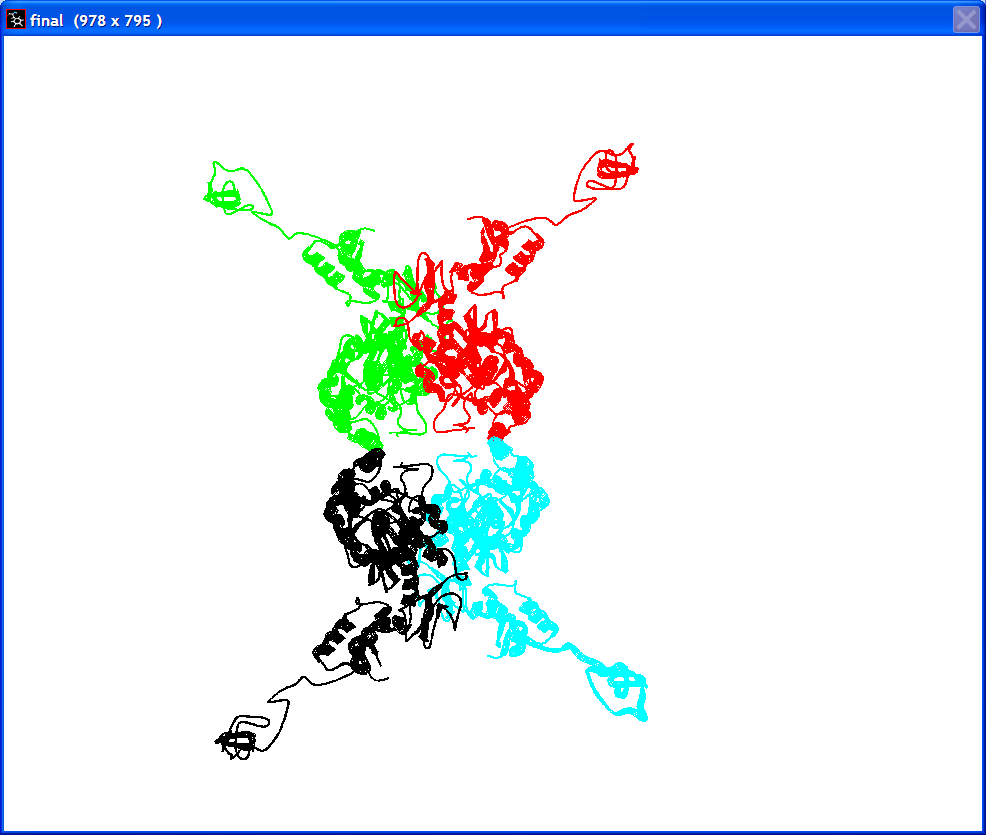


**Figure S9.** Estimation of S values, R_g_ values and Rh values of likely GACwt structures in solution. The values were calculated using the program HYDROPRO [[2](#_ENREF_2)].


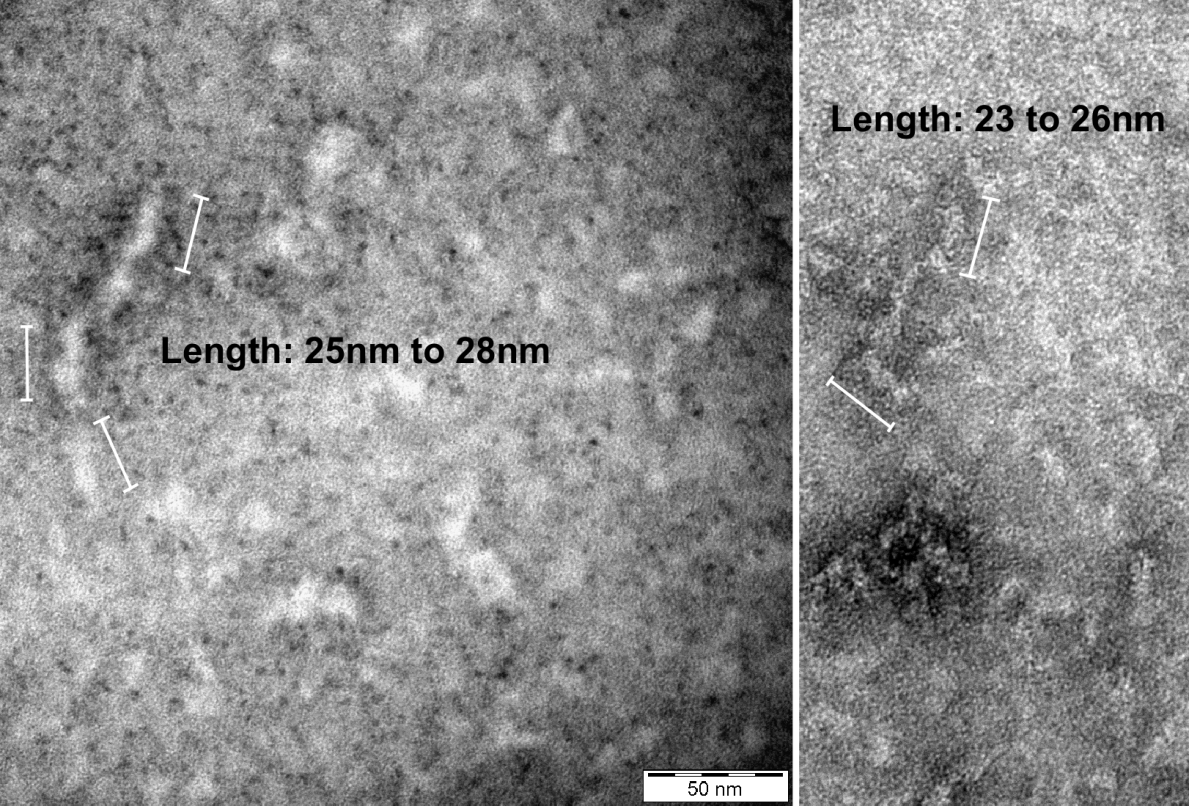


**Figure S10:** **TEM images of glutaminase C.** TEM images were recorded of GACwt at the concentration 0.68 μM. The staining method has been previously described [[3](#_ENREF_3)]. A scale bar is shown in the right corner of the image. The observed sizes of oligomers range from 230 to 280 Å. The images were recorded at Cornell Center for Material Research facility. It is expected that the maximum distance is variable since the protein system is flexible. For the calculated tetramer model, maximum distance = 250 Å. The images show that high oligomers, such as octamers, grow in an elongated direction.


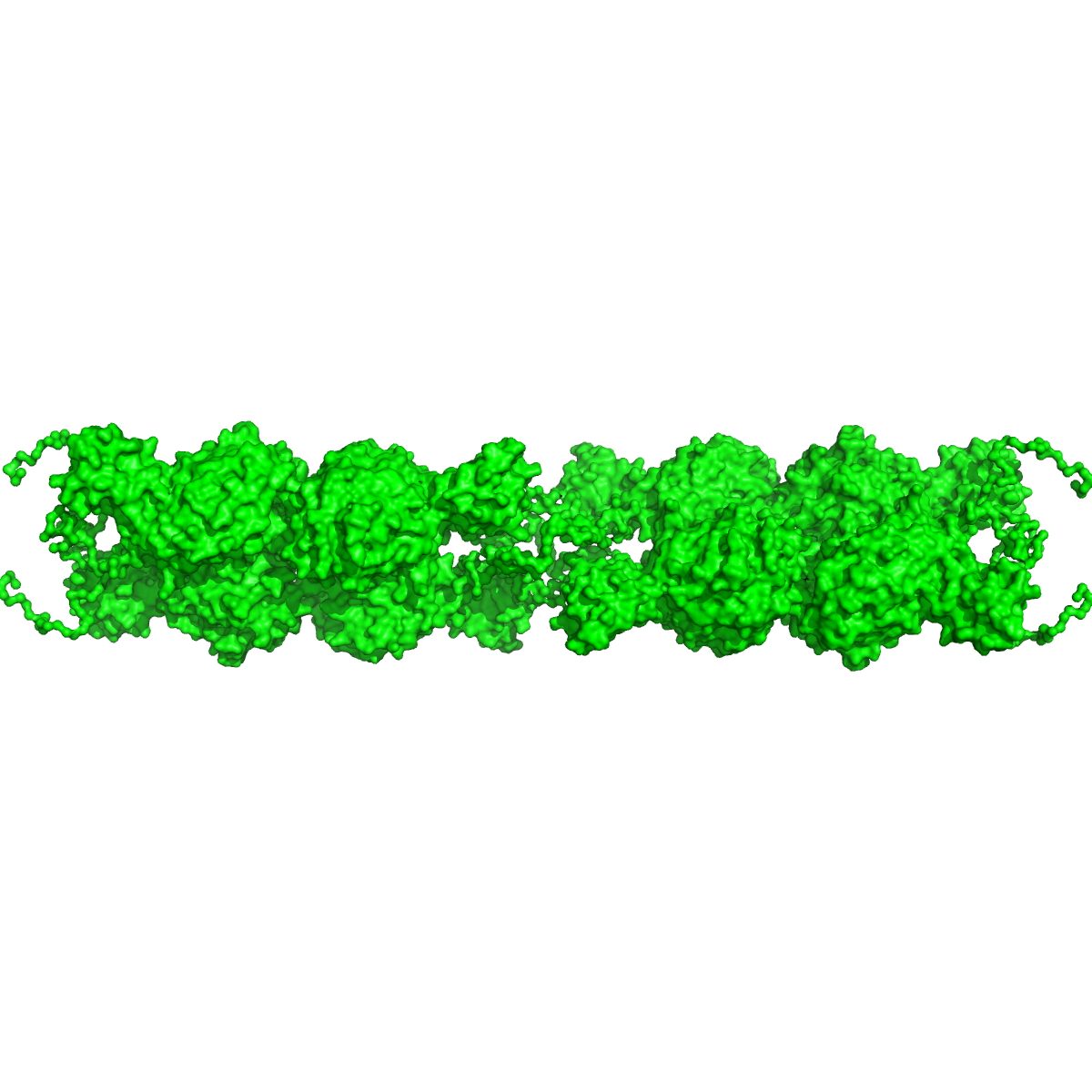
**a)**


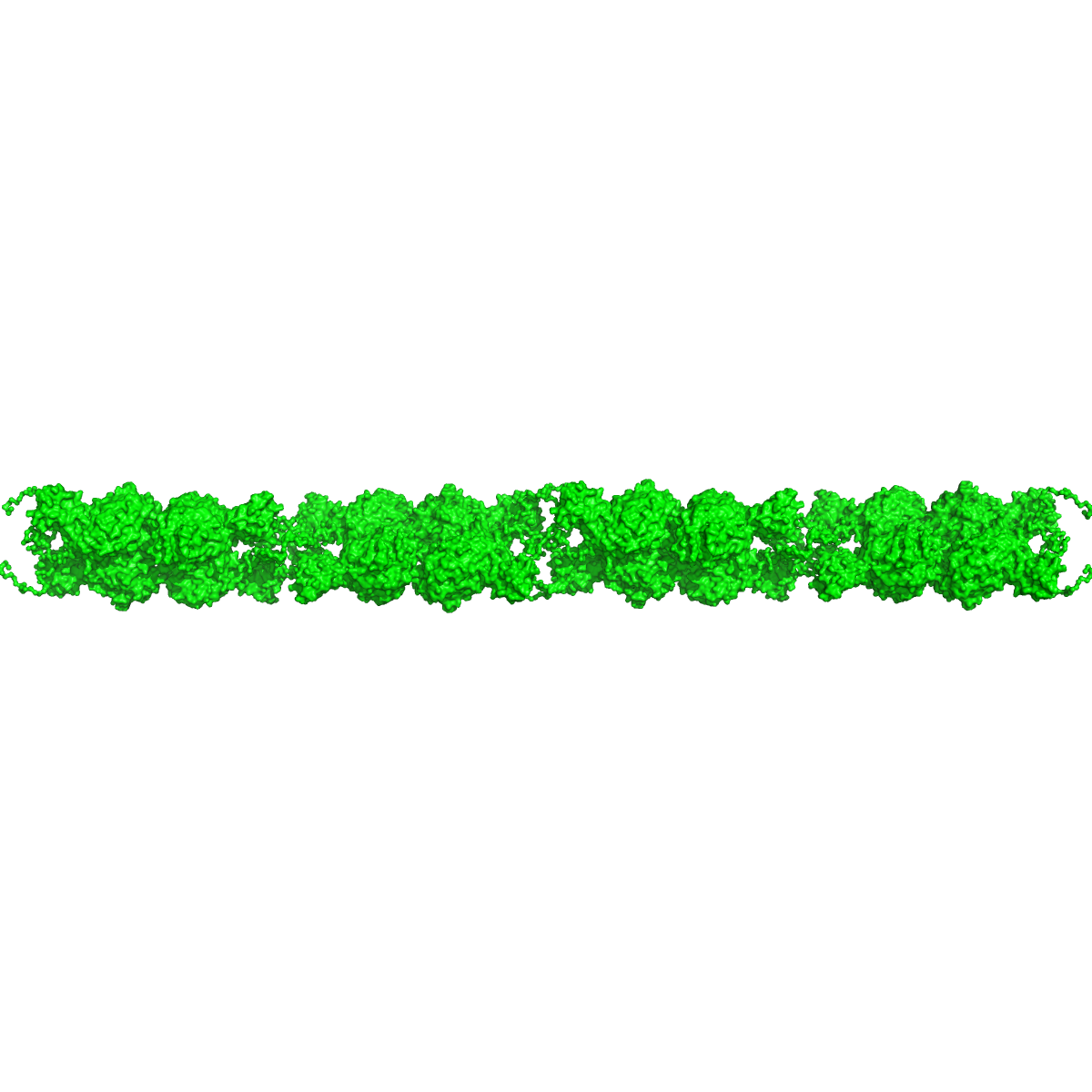
**b)**

**Figure S11:** **Higher oligomers.** Representations of the **a)** manually built octamer and the **b)** manually built 16mer. The structures were built using a tetramer in a relatively compact conformation generated by EOM. The tetramers were translated along the longest axis by 164 Å. All manipulations were carried out in Pymol [[4](#_ENREF_4)].

** Figure S12: EOM and OLIGOMER fits.** **a)** EOM fit to the experimental data for GACwt concentrations series at three concentrations. **b)** EOM fit to the experimental data for GACΔC concentrations series at three concentrations. **c)** OLIGOMER fit to the experimental data for GACwt concentrations series at three concentrations. **d)** OLIGOMER fit to the experimental data for GACΔC concentrations series at three concentrations. **e)** OLIGOMER fit to the experimental data for GACwt inorganic phosphate titration series at four concentrations. **f)** OLIGOMER fit to the experimental data for GACΔC Pi titration series at four concentrations.


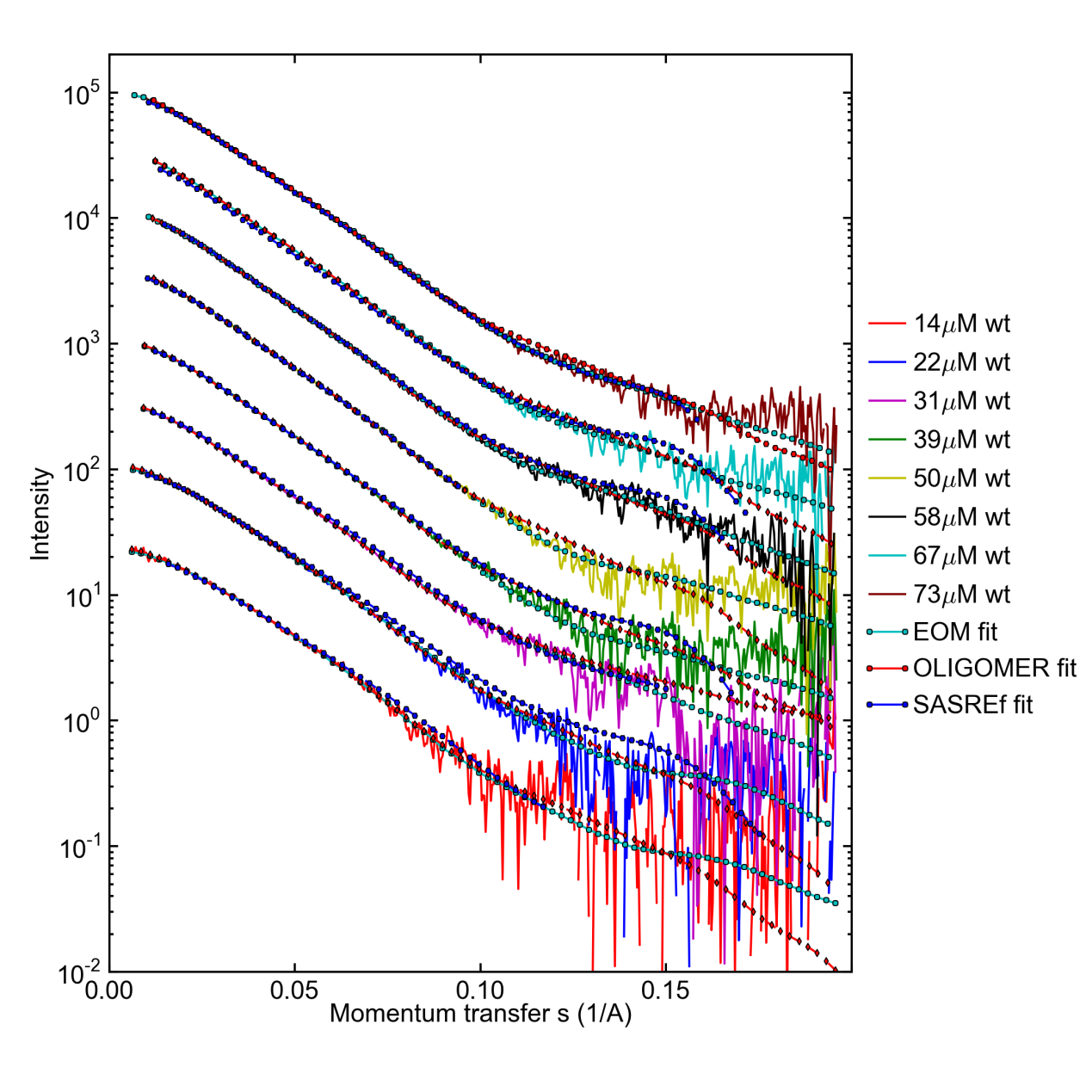


**Figure S13: EOM, OLIGOMER and SASREFMX fits.** EOM fit, OLIGOMER fit and SASREFMX fit to the experimental data for GACwt concentration screen. Fit curve for the three programs are plotted for each concentration curves to enable a comparison of the obtained fits for the programs.


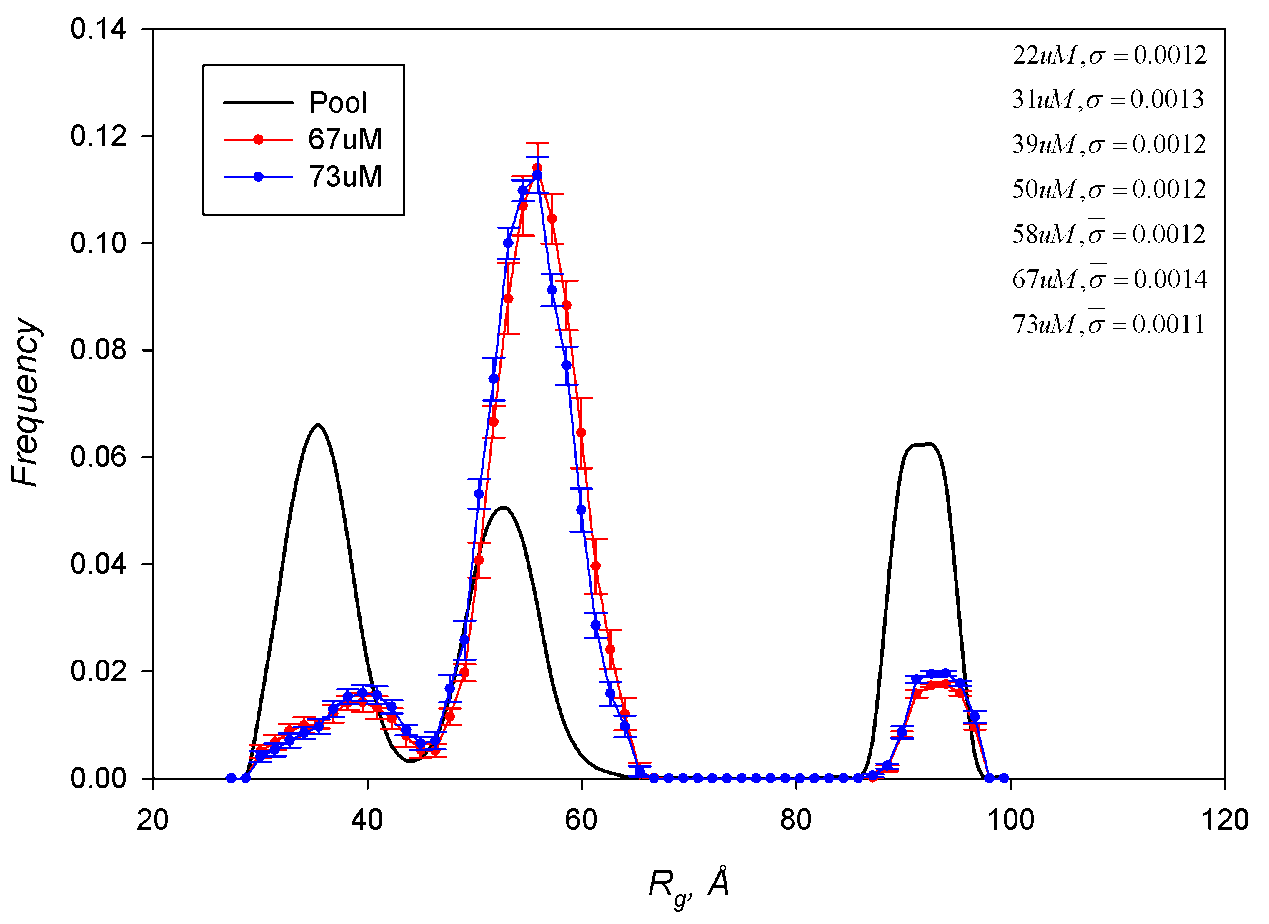


**Figure S14.** EOM analysis of GACwtCs. The plot shows the average distributions, out of 10 repetitions of the genetic algorithm, for constrains with the lowest (73uM, blue) as well as the largest (67uM, red) standard deviation.

**
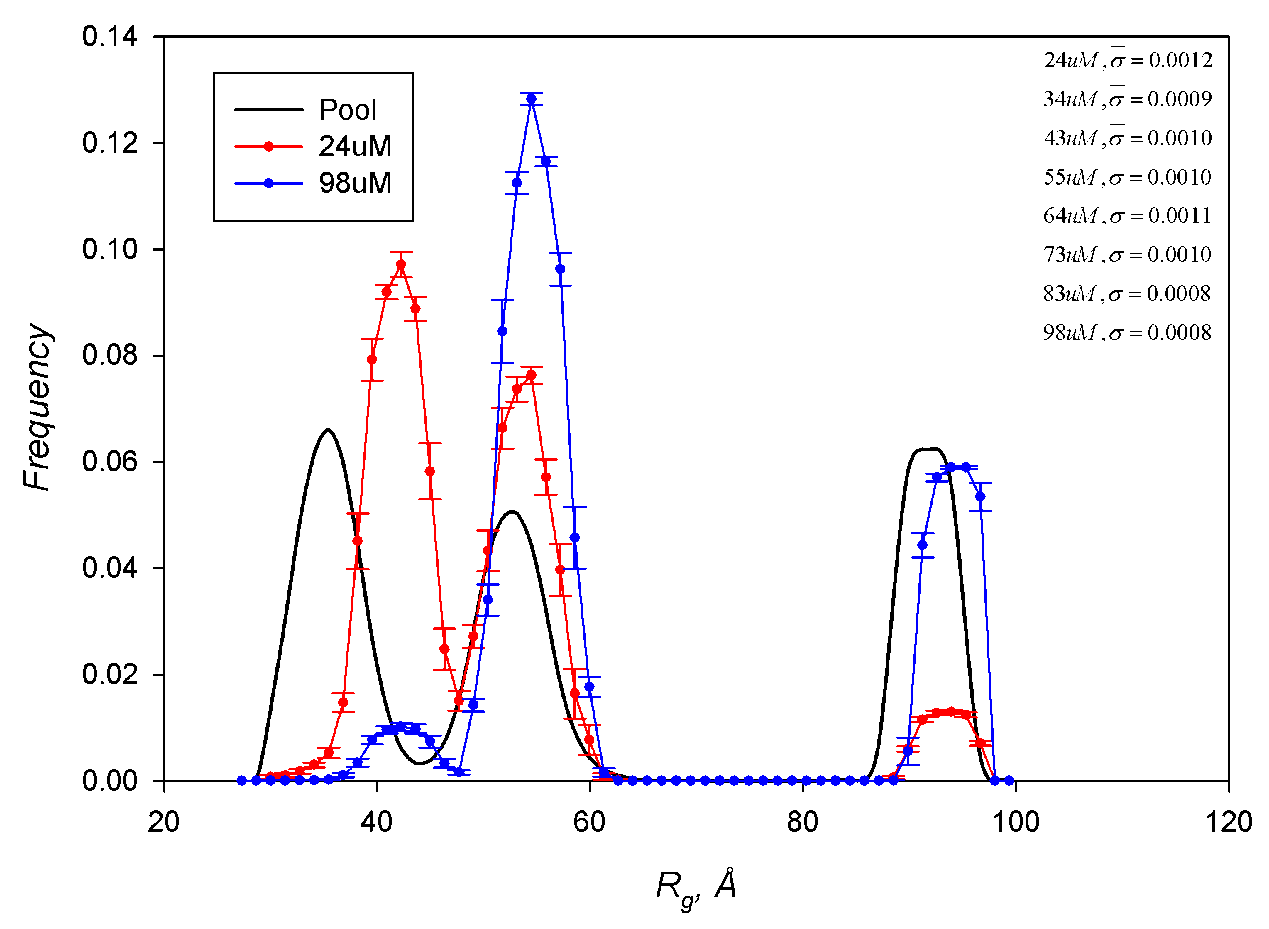
**

**Figure S15**. EOM analysis of GACΔC concentration screen. The plot shows the average distributions, out of 10 repetitions of the genetic algorithm, for constrains with the lowest (98uM, blue) as well as the largest (24uM, red) standard deviation.


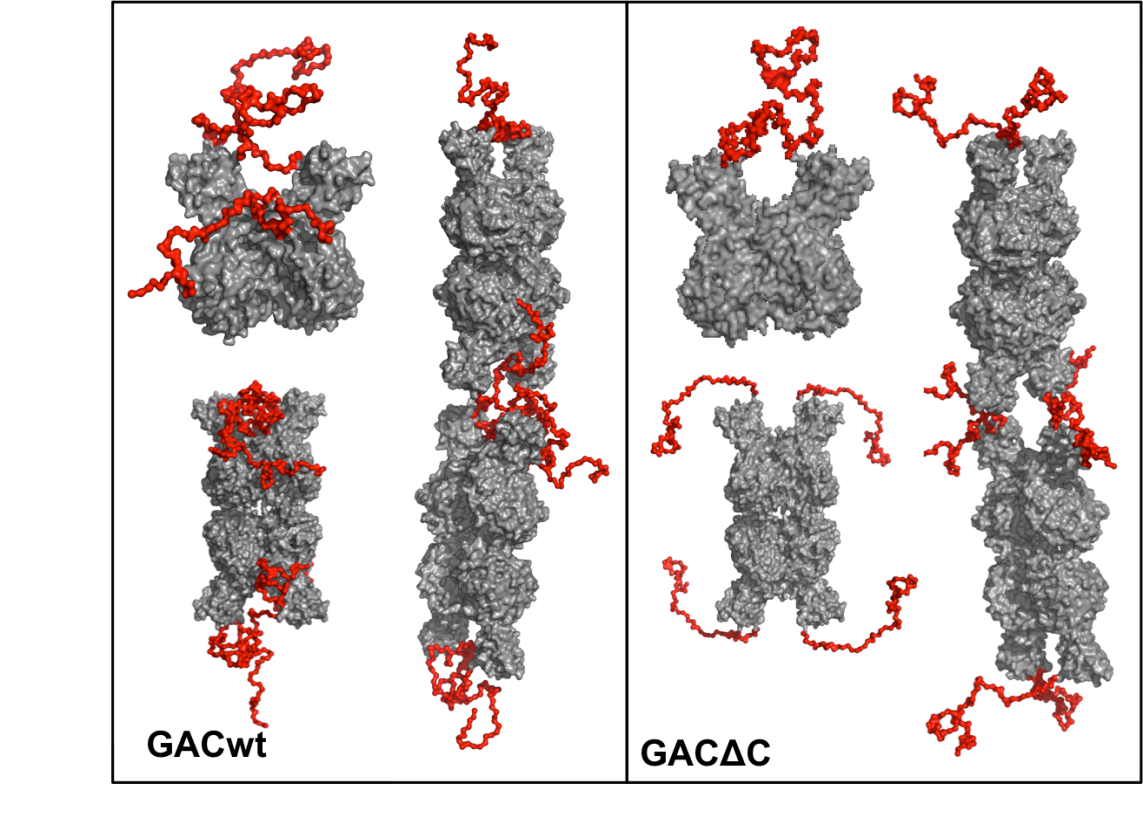


**Figure S16: EOM generated structures.** Selected EOM generated structures of dimers, tetramers and octamers for both GACwt and GACΔC. The structures were selected among those found by EOM program to give the best fit to the experimental data.

| **Vol. fractions of Dimers Tetramers Octamer** | | | | | | | | |
| --- | --- | --- | --- | --- | --- | --- | --- | --- |
|  |  | **Chi** | **Variant 1** | **Variant 2** | **Variant 3** | **Variant 1** | **Variant 2** | **Variant 1** |
| **Prot. uM** | 14 | 1.25 | 0 ± 0.00 | 0.3 ± 0.01 | 0 ± 0.00 | 0.7 ± 0.01 | 0 ± 0.00 | 0 ± 0.00 |
|  | 22 | 1.43 | 0 ± 0.00 | 0.1 ± 0.02 | 0.2 ± 0.03 | 0.7 ± 0.01 | 0 ± 0.01 | 0 ± 0.00 |
|  | 31 | 1.76 | 0 ± 0.00 | 0.1 ± 0.01 | 0.1 ± 0.01 | 0.6 ± 0.06 | 0.2 ± 0.07 | 0 ± 0.00 |
|  | 39 | 1.69 | 0 ± 0.00 | 0 ± 0.00 | 0.2 ± 0.01 | 0.8 ± 0.04 | 0 ± 0.00 | 0 ± 0.00 |
|  | 50 | 1.89 | 0 ± 0.00 | 0 ± 0.00 | 0.1 ± 0.00 | 0.8 ± 0.01 | 0 ± 0.00 | 0 ± 0.01 |
|  | 58 | 1.69 | 0.1 ± 0.02 | 0 ± 0.00 | 0 ± 0.01 | 0.8 ± 0.01 | 0 ± 0.00 | 0 ± 0.01 |
|  | 67 | 2.04 | 0.1 ± 0.02 | 0 ± 0.00 | 0 ± 0.01 | 0.8 ± 0.01 | 0 ± 0.00 | 0 ± 0.01 |
|  | 73 | 2.29 | 0.1 ± 0.01 | 0 ± 0.00 | 0 ± 0.01 | 0.8 ± 0.01 | 0 ± 0.00 | 0.1 ± 0.01 |
| **Pi mM** | - 0 | - 1.35 | - 0.1 ± 0.01 | - 0 ± 0.00 | - 0 ± 0.00 | - 0.6 ± 0.04 | - 0.2 ± 0.01 | - 0.1 ± 0.01 |
|  | - 12 | - 1.53 | - 0 ± 0.00 | - 0.1 ± 0.00 | - 0 ± 0.00 | - 0.8 ± 0.01 | - 0 ± 0.00 | - 0.1 ± 0.01 |
|  | - 25 | - 1.52 | - 0 ± 0.00 | - 0.1 ± 0.00 | - 0 ± 0.00 | - 0.8 ± 0.01 | - 0 ± 0.00 | - 0 ± 0.01 |
|  | - 50 | - 2.10 | - 0 ± 0.00 | - 0 ± 0.00 | - 0 ± 0.00 | - 0.8 ± 0.01 | - 0 ± 0.00 | - 0.2 ± 0.01 |
|  | - 60 | - 2.57 | - 0 ± 0.00 | - 0 ± 0.00 | - 0 ± 0.00 | - 0.8 ± 0.01 | - 0 ± 0.00 | - 0.2 ± 0.01 |
|  | - 80 | - 4.61 | - 0 ± 0.00 | - 0 ± 0.00 | - 0 ± 0.00 | - 0.6 ± 0.01 | - 0 ± 0.00 | - 0.4 ± 0.01 |

**Table S2: OLIGOMER parameters and results for glutaminase C wild type construct.** Three different dimer conformations (variant 1, 2 and 3 all with different R_g_ values (please refer to Materials and Methods), two tetramer conformations (variant 1 and 2 with different R_g_ values (please refer to Materials and Methods) and one octamer conformation (variant 1) were used for the analysis.

| **Vol. fractions of Dimers Tetramers Octamer** | | | | | | | | |
| --- | --- | --- | --- | --- | --- | --- | --- | --- |
|  |  | **Chi** | **Variant 1** | **Variant 2** | **Variant 3** | **Variant 1** | **Variant 2** | **Variant 1** |
| **Prot. (u)M** | 24 | 1.33 | 0 ± 0.00 | 0.3 ± 0.02 | 0 ± 0.03 | 0.6 ± 0.02 | 0 ± 0.00 | 0.1 ± 0.01 |
|  | 34 | 1.53 | 0 ± 0.00 | 0.1 ± 0.02 | 0.2 ± 0.02 | 0.6 ± 0.01 | 0 ± 0.00 | 0.1 ± 0.01 |
|  | 43 | 2.63 | 0 ± 0.00 | 0 ± 0.00 | 0.1 ± 0.00 | 0.6 ± 0.01 | 0 ± 0.00 | 0.3 ± 0.01 |
|  | 55 | 4.26 | 0 ± 0.00 | 0 ± 0.00 | 0.1 ± 0.00 | 0.5 ± 0.01 | 0 ± 0.00 | 0.4 ± 0.00 |
|  | 64 | 4.17 | 0 ± 0.00 | 0. ± 0.01 | 0. ± 0.01 | 0.5 ± 0.01 | 0 ± 0.00 | 0.5 ± 0.00 |
|  | 73 | 5.09 | 0 ± 0.00 | 0 ± 0.00 | 0 ± 0.00 | 0.5 ± 0.00 | 0 ± 0.00 | 0.5 ± 0.00 |
|  | 83 | 6.04 | 0 ± 0.00 | 0 ± 0.00 | 0 ± 0.00 | 0.4 ± 0.00 | 0 ± 0.00 | 0.6 ± 0.00 |
|  | 98 | 7.13 | 0 ± 0.00 | 0 ± 0.00 | 0 ± 0.00 | 0.3 ± 0.00 | 0 ± 0.00 | 0.7 ± 0.00 |
| **Pi mM** | - 0 | 1.46 | - 0 ± 0.00 | 0.1 ± 0.01 | 0 ± 0.02 | 0.6 ± 0.01 | 0 ± 0.00 0.001 | 0.2 ± 0.01 |
|  | - 12 | 1.78 | - 0 ± 0.00 | 0.1 ± 0.00 | 0 ± 0.00 | 0.7 ± 0.01 | 0 ± 0.00 | 0.2 ± 0.01 |
|  | - 25 | 2.05 | - 0 ± 0.00 | 0.1 ± 0.02 | 0.1 ± 0.02 | 0.6 ± 0.01 | 0 ± 0.00 | 0.3 ± 0.01 |
|  | - 50 | 2.30 | - 0 ± 0.00 | 0.0 ± 0.03 | 0 ± 0.03 | 0.2 ± 0.02 | 0 ± 0.00 | 0.8 ± 0.01 |
|  | - 60 | 1.91 | - 0 ± 0.00 | 0.1 ± 0.01 | 0 ± 0.00 | 0 ± 0.00 | 0 ± 0.00 | 0.9 ± 0.00 |
|  | - 80 | 2.70 | - 0 ± 0.00 | 0 ± 0.00 | 0 ± 0.00 | 0 ± 0.00 | 0 ± 0.00 | 1.0 ± 0.00 |
|  | - 100 | 13.0 | - 0 ± 0.00 | 0 ± 0.00 | 0 ± 0.00 | 0 ± 0.00 | 0 ± 0.00 | 1.0 ± 0.00 |

**Table S3: OLIGOMER parameters and results for glutaminase C construct with**

**truncated C-terminal.** Three different dimer conformations (variant 1, 2 and 3 all with different R_g_ values (please refer to Materials and Methods)), two tetramer conformations (variant 1 and 2 with different R_g_ values (please refer to Materials and Methods) and one octamer conformation (variant 1) were used for the analysis.


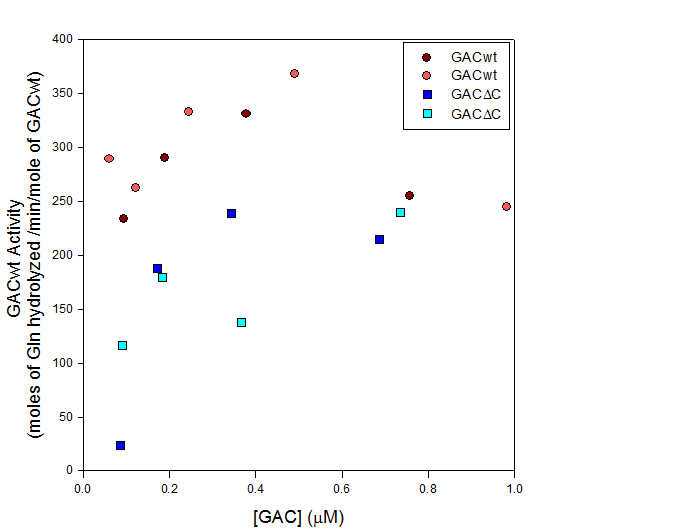


**Figure S17: Protein concentration dependence of the activity of glutaminase C wild type construct and C-terminal truncated construct.** The data shown are a composite of two independent experiments.


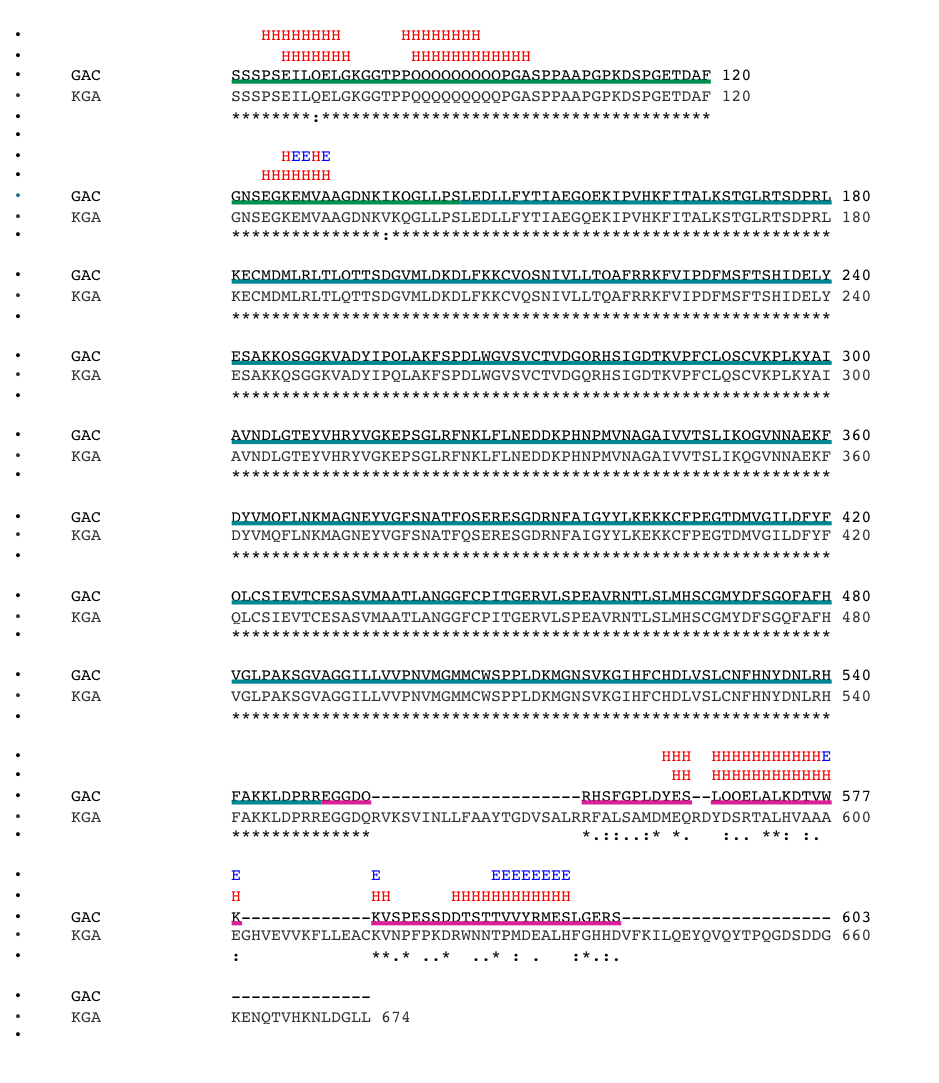


**Figure S18**. Sequence alignment of mouse GAC and rat KGA, using the NCBI reference sequences NP_001106854 and AAA41237.1 respectively. The alignment was done using the ClustalW program [[5](#_ENREF_5)]. The KGA sequence is shown in gray and GAC sequence is shown in black. The part of GAC sequence with known crystal structure is underlined with turquoise color, the N-terminal is underlined with green color and the C-terminal is underlined with pink color. The results of the secondary structure prediction for N- and C-termini are illustrated in the figure: Predicted extended regions are shown in blue E letters, predicted helical regions are shown in red H letters. Internet services were used for the predictions [[6](#_ENREF_6),[7](#_ENREF_7)]. The tool used for the upper line of structure prediction does not include extended regions as a possible as does the tool used for the prediction shown in lower line.

1. Toft KN, Vestergaard B, Nielsen SS, Snakenborg D, Jeppesen MG, et al. (2008) High-Throughput Small Angle X-ray Scattering from Proteins in Solution Using a Microfluidic Front-End. Analytical Chemistry 80: 3648-3654.

2. Ortega A, Amorós D, García de la Torre J (2011) Prediction of Hydrodynamic and Other Solution Properties of Rigid Proteins from Atomic- and Residue-Level Models. Biophysical Journal 101: 892-898.

3. Olsen BR, Torgner, I A, Christensen, T B, Kvamme, E. (1972) Ultrastructure of pig renal glutaminase. Evidence for conformational changes during polymer formation. J Mol Biol 74: 239-251.

4. Schrodinger, LLC (2010) The PyMOL Molecular Graphics System, Version 1.3r1.

5. Thompson JD, Higgins DG, Gibson TJ (1994) CLUSTAL W: improving the sensitivity of progressive multiple sequence alignment through sequence weighting, position-specific gap penalties and weight matrix choice. Nucleic Acids Research 22: 4673-4680.

6. Chou PY, Fasman GD (1974) Conformational parameters for amino acids in helical, β-sheet, and random coil regions calculated from proteins. Biochemistry 13: 211-222.

7. ( ) SCRATCH Protein Predictor [www.scratch.proteomics.ics.uci.edu](http://www.scratch.proteomics.ics.uci.edu). Accessed June 2013.
